# Supplementary material for: Effect of a 90 g/day low-carbohydrate diet on glycaemic control, small, dense low-density lipoprotein and carotid intima-media thickness in type 2 diabetic patients: An 18-month randomised controlled trial
Source: PLoS One. 2020 Oct 5;15(10):e0240158. doi: 10.1371/journal.pone.0240158 (PMC7535044; doi:10.1371/journal.pone.0240158)
Supplement: S1 File — (PDF) [file pone.0240158.s002.pdf]

低醣飲食在減少第二型糖尿病患者多重用藥的效果

The effectiveness of low carbohydrate diet in reducing polypharmacy for patients with type 2 diabetes mellitus

一、研究計畫中英文摘要：請就本計畫要點作一概述，並依本計畫性質自訂關鍵詞。

（1） 計畫中文摘要。（五百字以內）

糖尿病的飲食控制，傳統上大多建議碳水化合物(醣類)佔 50-60%的攝取量，脂肪佔 30%以下，但醫學上其實並沒有足夠的證據，包括：系統性回顧(systematic review)或巨集分析(meta-analysis)，去支持這樣的建議。有越來越多的證據，支持低醣飲食的效果以及方便性，但缺乏長期有效的研究。可能缺乏有效又兼顧持久性的低醣限制；以及統計上，沒有同時考量低醣飲食藥物減少或傳統飲食藥物增加的影響。

因此，本研究以隨機對照研究，選擇門診 20 到 80 歲的第二型糖尿病、糖化血色素≧7.5 者，針對不同性別、身體質量指數分層(BMI≦24, >24；男性與女性)，樣本估計為 120 人，以低醣飲食介入(每天醣類攝取< 90 克，不限卡路里) 。藥物由醫師依研究設定原則調整。對照組: 營養師傳統卡路里限制飲食(calorie-restricted diet)：醣類：50-60%，蛋白質：1.0-1.2 克/公斤 (<20%) 及脂肪≦30%衛教， 第一線 metformin ；第二線由醫師自行調整。追蹤 18 個月，初級結果(primary outcome)為藥物增減種類及劑量，以藥物效果計分(medication effect score calculation) 看糖尿病多重藥物減少的效果及血糖控制狀況(含飯前、飯後血糖及糖化血色素)。次級結果(secondary outcome)為生化檢查、身體測量、代謝指標、功能指標、 動脈硬化、糖尿病困擾指數(The 17-item version of the Chinese Diabetes Distress Scale ,CDDS-17)等全方位的效果，並提供提供適當可行的飲食方案，以促進糖尿病的良好控制及減少多重用藥。

（2）　計畫英文摘要。(五百字以內)

Most of the Diabetic Associations recommend a carbohydrate intake of 50-60% of total energy intake, total fat intake ≦30% of energy. However, there is insufficient evidence, including systematic review or meta-analysis, to justify these recommendations. More and more evidence supports the effect and convenience of low carbohydrate diet, but the long-term evidence is insufficient. The lack of evidence could be due to inadequate carbohydrate restriction, non-compliance or the adjustment of hypoglycemic agents mask the real effect of diet control.

Our randomized controlled trial is designed for age 20 to 80 type II Diabetes Mellitus outpatients with HbA1C≧7.5. They will be stratified according to sex and body mass index(BMI) (BMI≦24, >24, man or woman).

The sample size is estimated to be 120 in total. The experimental group is low carbohydrate diet group (carbohydrate less than 90g/day without limitation of caloric intake). The control group is traditional caolorie-restricted diet: carbohydrate 50-60%, protein1.0-1.2 g/kg (<20%) and fat≦30%). The hypoglycemic agents are adjusted by the physicians according to the study rule. The follow-up time is 18 months.The primary outcome is the adjustment of medication (types or dosage), the scores of medication effect score calculation and glycemic control (HbA1c, fasting glucose and postprandil glucose).

The secondary outcomes are biochemical change, metabolic change,physical assessment, functional assessment, atherosclerosis assessment and Diabetes Distress Scale (The 17-item version of the Chinese Diabetes Distress Scale,CDDS-17) . Our aim is to testthe effectiveness of low carbohydrate diet to improve the diabetic control and reduce the polypharmacy.

二、研究計畫內容：

(一) 研究計畫之背景及目的。

背景:

糖尿病是心血管疾病的主要危險因子，和高血壓、高脂血症、缺血性心臟病、心臟衰竭、腦血管疾病、周邊血管疾病等共病；而這些血管性疾病，藉由疾病本身，或是和失智症、憂鬱症等相關，變成是老化過程中，動脈硬化疾病相關衰弱症的元兇，而衰弱症則和失能、住院、照護需求及死亡相關。除了癌症相關的衰弱症以外，血管性衰弱症，應該是要回歸主要慢性病如糖尿病的防治，才能從根本預防老化。因此，糖尿病的治療良好，對老化社會，是重要的預防指標。

雖然對於第二型糖尿病的飲食控制在世界各學會多有規範，如：
British Diabetes Association, European Association for the Study of Diabetes (EASD), American Diabetes Association(ADA), American Heart Association, Canadian Diabetes Association,International College of Nutrition, groups from SouthAfrica and Japan, and the National Cholesterol Education Panel(Adult Treatment Panel 3)傳統上大多都建議碳水化合物(醣類)佔 50-60%的攝取量，脂肪佔 30%以下，但醫學上其實並沒有足夠的證據，包括：系統性回顧(systematic review)或巨集分析(meta-analysis)，去支持這樣的建議[1]。

我們可以從 ADA 發表的聲明一窺端倪，ADA 在 2007 年的 Standards of Medical Care in Diabetes 不建議低醣飲食是因為沒有足夠的證據支持長期的效果以及心血管疾病危險性是否上升仍不明[2]，但在 2008 年 ADA 改了論述[3]，對於過重的糖尿病患者或非糖尿病患者，他們建議可使用短期的低脂肪飲食或是低碳水化合物(醣)飲食幫助減肥(2008 年為 1 年，至 2011 年已提升為 2 年[4])。

事實上，在 2009 年 ADA 出版糖尿病生活(Life With Diabetes) 就已記載「醣分、蛋白質及脂質均含卡路里，但只有糖份直接影響血糖」(Carbohydrate, protein, and fat contain calories. Only Carbohydrates directly affect blood glucose levels) [5] 。2013 年 ADA 甚至去除了一天需要 130 克的碳水化合物這段話[6]。而在 2014 年 1 月的 ADA 發表的 Diabetes Care，提到低油飲食在過去研究包括巨集分析和系統性回顧，並無法達到良好的血糖控制以及減少心血管疾病風險[7]。Standards of Medical Care in Diabetes—2014 也提到對於目前盛行的幾種飲食法包括 1.地中海性飲食 (Mediterranan Style) 2. 素食(Vegetarian and vegan) 3. 低脂(Low fat) 4. 低碳水化合物(醣) (Low carbohydrate) 5. DASH (Dietary Approaches to Stop Hypertension)(少油少甜多蛋白少紅肉多纖維低鈉)， ADA 並無法提出最理想的飲食法，在 2015 也是一樣。

雖然低碳水化合物(醣)飲食並非主流，但越來越多證據發現這樣的治療危險性低並容易遵從[2]。但其中的低碳水化合物(醣)飲食，依照最新的建議[8]：分類為：(1)極低醣質酮生成飲食(very low-carbohydrate ketogenic diet, VLCKD)，醣質的攝取為每天 20-50 克，或小於 10% 每天 2000 卡的飲食；(2)低醣質飲食：醣質的攝取為每天小於 130 克，或小於 26% 每天總能量；(3)中醣質飲食：醣質的攝取為每天飲食的 26-45% 每天總能量；(4) 高醣質飲食：醣質的攝取為每天

飲食的＞45% 每天總能量。

最近的一篇系統性回顧[1]比較了不同種飲食對糖尿病的控制，結果發現低醣飲食、低升糖指數飲食、地中海飲食和高蛋白飲食對血糖的控制都比對照組好 ，低醣飲食和地中海飲食的體重減輕最明顯。

所回顧的文獻[9-17]大多以肥胖者為對象[9-11, 14-17]，有三篇包含 30-40%的糖尿病病人[9.10.16]有四篇是肥胖的糖尿病病人[11,14,15, 17]，只有兩篇不是針對肥胖者，包括一篇是飲食控制的糖尿病病人[12]及一篇是一般糖尿病病人[13]。在不純粹之糖尿病患者研究中，6 個月的研究[9]，對體重及血糖、三甘油脂有顯著改善；但在一年中的研究[10]卻缺乏顯著的體重改善，可能低醣飲食較難維持、有較高的流失率(drop out)，也可能因為對照組的飲食控制效果不錯。另一篇則是以低醣飲食和低脂飲食併用減肥藥 Orlistat 相比[16]，一年的研究，兩者沒有顯著差別，而且本篇的醣類攝取，在 48 週時還能維持 62 克/天(15%卡路里)，和其他研究相比算是有不錯的遵從性。

在糖尿病患者的研究中，使用約 40% 醣類的研究[12]，在以飲食控制的糖尿病病人，半年追蹤，和傳統糖尿病飲食，在血糖控制上並無明顯差別。在另一日本的兩年非隨機對照研究，使用約 40% 醣類，有較好的血糖控制、體重、總膽固醇及低密度脂蛋白，並可減少血糖藥的種類跟劑量，特別是磺醯尿素 Sulfonylurea[13]。在過重糖尿病病人，原先設計的 20-30 克醣質控制，只有前三個月在體重及醣化血色素有效，到一年後飲食無法持續，醣質 77±44 克(24%卡路里)，兩組沒有差別[14]。

最近的隨機對照研究(總人數 24 人)，在日本針對糖尿病血糖控制糖化血色素在 6.9 到 8.4 者，將醣分控制每天 70-130 克，半年可降低糖化血色素約 0.5% (P=0.03) [18]。

對於腎功能，2013 年 8 月在 Diabetes Care[19]發表的一篇針對三種不同飲食(低醣、地中海飲食、低脂飲食)的隨機對照研究中，對象人數為 318 人，收案標準為慢性腎臟病(chronic kidney disease, CKD) stage III 以下病人，在兩年中，發現不管是哪種飲食方法，在達到減重效果以外，對慢性腎臟病 CKD stage I,II,或 III 的病人，eGFR 都能夠提升。表示低醣飲食對腎功能的影響地中海飲食及低脂飲食並無顯著差異。

對於心血管疾病因子如血脂肪,CRP 的影響，在 2012 年 11 月於 Obes Rev 發表一篇對過去低醣飲食的系統性回顧[20]，發現在體重,身體質量指數(BMI),血壓,腹圍,三甘油脂(TG), HbA1c, insulin, CRP 的下降都有達到統計顯著, HDL 的上升也達到統計顯著，而 LDL 和肌酸酐並無明顯變化。

對於一些其他的代謝性指標，顯示低醣飲食在三個月的血液中可降低 hs-CRP 及增加 total adiponectin [21]。

較長期的隨機對照研究，包括：比較高蛋白低醣飲食和高醣類飲食對第二型過重或肥胖糖尿病病人，追蹤一年在醣化血色素及脂質代謝上並無明顯優勢

[22]，但所採用的高蛋白低醣飲食為蛋白質為 30%、醣質為 40%；高醣飲食則為蛋白質為 15%、醣質為 55%，對象為肥胖或過重糖尿病患者。可能兩組人都有行為改變，或是自我報告飲食狀況及可選擇食物之限制，總人數 93 人較少但有符合研究設計的估算人數，但實際的醣質數無法由研究中看出。兩年的研究，和上述同樣的低醣配方[23]，人數雖多達 419 人，根據研究所給予的每日卡路里數(2000 千卡/天)推算為每日 200 克醣質，結果沒有顯著差別，不能排除須要更低的醣質限制。另一篇兩年的研究結果[24]，對象也是肥胖或過重糖尿病患者，雖然採用較低的醣質(20%)，根據研究所給予的男女每日卡路里數(1800 及 1600 千卡/天)推算為：男性每日 90 克醣質，女性每日 80 克醣質，雖然飲食記錄遵從性很好，但研究人數只有 61 人，在體重、醣化血色素、胰島素及高密度膽固醇並無差別。

在另一個小型糖尿病及非糖尿病各半的研究中(人數 26 人)[25]，醣質限制為每日 40 克，在三個月時可見體重及糖化血色素顯著減少，但研究後觀察兩年，飲食控制沒有繼續支持的狀態，兩年後並沒有顯著的好處。

文獻 8 針對低醣飲食做了系統性的回顧，整理為以下 12 點：

1. 糖尿病最明顯的特性是高血糖，而限制醣類攝取對降低血糖有最明顯的效果。證據: 在一前瞻性研究[26]中收入 102 個糖尿病病人及 261 個非糖尿病病人，分派到 VLCKD 及 low-calorie diet 組，在 24 週後發現平均血糖值、糖化血色素，在 VLCKD 均較 low-calorie diet 組好(HbA1c: VLCKD: 6.2%, low-calorie diet: > 7.5%)。
2. 在現今肥胖與 2 型糖尿病盛行的時代，攝取過多的熱量主要來自於碳水化合物的過多攝取。NHANES(National Health and Nutrition Examination Survey) 調查發現在男性碳水化合物所佔的熱量從 1974 年的 42%升高到 2000 年的 49%，女性則從 45%升到 52%，而 2 型糖尿病的發生率也越來越多，顯示可能有相關[27]。研究顯示[28]持續刺激胰島素分泌，會刺激身體傾向三酸甘油酯(TG)以及其他含富含 TG 之脂蛋白的合成(anabolic state)。現在也相信肝臟以及胰臟的脂肪堆積，會增加 VLDL 的製造進而將脂肪帶到胰臟，對於 beta-cell 的功能會有不良的影響[29]。
3. 限醣飲食對於糖尿病病人的血糖控制能改善的原因並非只來自於體重減輕。證據：研究顯示給予 2 型糖尿病某種程度的限醣飲食(30% carbohydrate (CHO), 30% protein, 40% fat, (30:30:40) (LoBAG30)，實際上並沒有達到前述的 low carbohydrate diet 的定義(<130g/d or <26% total energy))，在十週後雖然體重並沒有改變，但血糖控制的確有良好效果[30]。
4. 雖然限醣飲食的好處不只來自於減肥，但對於減肥，目前限醣飲食為最有效的做法。英國某研究[31]收了 26 個志願者(其中 13 位為糖尿病)分派到限醣飲食(40 g/d carbohydrate)及英國傳統的健康飲食組中，追蹤三個月，發現限醣飲食體重減輕較多(6.9 kg 對 2.1 kg)。Women's Health Initiative Dietary Modification Trial 針對約 480000 個停經婦女做低脂飲食的研究[32]，顯示低脂飲食對減肥效果有限並容易復胖。值得注意的是以往流行的 Atkins diet(為限醣飲食)[33,34]，針對攝取熱量並無限制，只要求不攝取碳水化合物，因為大多數人相信脂肪和蛋白質較有飽足感，所以可以減少總熱量的攝取。
5. 限醣飲食的遵從性並不亞於傳統的低脂飲食或熱量限制飲食，甚至更易遵從[35-37]。原因可能在於計算熱量太麻煩以及蛋白質及脂肪能夠增加飽足感以及減少血糖波動，對於糖尿病患者來說更可以減少他們的藥物使用及胰島素

注射。

6. 研究顯示以蛋白質攝取來替代醣類攝取對於身體的組成(如 Fat-free body mass)以及其他健康指標均有良好影響[38-40]

7. 飲食中脂肪的攝取量和心血管疾病的風險並無相關。

8. 食物中的碳水化合物比起食物中的脂肪，更會影響血液中的飽和脂肪酸。血液中飽和脂肪酸的濃度與心血管疾病的危險性及胰島素阻抗性息息相關[51]。數篇研究[52-54]顯示食物中的飽和脂肪酸與血液中的飽和脂肪酸濃度並無相關，反而與碳水化合物較相關。

9. 在 2 型糖尿病病人，最佳預測小血管和大血管併發症的因子，是 HbA1c。UKPDS 針對 5102 個新診斷的 2 型糖尿病病人做研究，隨著每減少 1% 的 HbA1c，心肌梗塞事件的發生率可減少 14%，而小血管併發症的危險性可減少 37%[55-57]。

10. 限醣飲食對於減少血液中的 TG 及升高 HDL，是最有效的方法。研究顯示低 GI(glycemix index)指數的飲食可增加血液的 HDL 濃度，而這個效果在限醣飲食又更為明顯[11,58-59]。雖然傳統上認為總膽固醇及 LDL 濃度與心血管疾病發生危險性相關，但近來越來越多研究[60-62]顯示他們並不是好的預測因子，更好的預測因子可能包括 apolipoprotein(apo)B， 總膽固醇對 HDL 的比值， small dense LDL，以及 apoB 對 apoA1 的比值。也有研究[63]顯示 TG 對 HDL 的比值與 smaller dense LDL 的濃度呈現相關。

11. 2 型糖尿病病人在執行限醣飲食之後，可以減少藥物使用甚至不需要使用藥物。1 型糖尿病病人通常也可減少胰島素的施打。[64-70]

12. 限醣飲食比起其他飲食控制配上積極的藥物血糖控制，危險性較低。著名的 ACCORD 試驗[71]顯示積極血糖控制比起較不積極血糖控制，有較高心血管死亡率及較多低血糖發生率。限醣飲食從根本著手，減少藥物的使用，不僅能減少藥物費用支出，更能讓病人較好控制血糖。

同樣是亞洲國家的日本，最近也吹起糖質制限(=限醣飲食)的熱潮，在京都高雄病院的江部康二醫師，本身在罹患糖尿病後，針對糖尿病的本質進行反思及研究、系統性的整理文獻，以及在自己的病院對糖尿病患者進行糖質制限飲食指導後，發現效果非凡[72-73]，於是向整個日本醫療體系及社會宣導糖質制限飲食的概念，越來越獲得認同，其著作也成為日本暢銷書。日本各飲食業也紛紛跟進，開始設計低碳水化合物的飲食。

綜上所述，可見低醣飲食在 ADA 的指引中有逐漸被接納，也被認為是糖尿病的初步治療方針，但尚缺乏長期(一年以上)有效的證據；研究多限於過重或肥胖的糖尿病患者，沒有將一般體位或過瘦之糖尿病病人納入，不符合一般糖尿病照護原則；對於醣質攝取限制的量，要兼顧有效性及持久性，尚無定論，這也可能是缺乏長期有效證據的原因；治療中藥物的調整大多缺乏評估：低醣飲食可減少藥物或劑量、容易達成血糖控制，一般飲食經由增加藥物或劑量，達到血糖控制，所以，缺乏長期有效的證據，不能排除是在雙方藥物增減的差異下，達到共同有效的結果；但更長遠來說，過多的藥物達到血糖控制，增加醫藥花費、提早二度藥物失效的機會(secondary failure)，容易遵從的低醣飲食建議減少飲食控制的困難度，增進糖尿病的良好控制率，也是值得再研究之處。

目的

本研究希望藉由已有證據，提出適當有效的醣質限制，針對第二型糖尿病病人，做長期的追蹤，了解限醣飲食在血糖控制、血糖藥物之增減、生化指標、身體測量、代謝指標、功能指標、 動脈硬化、生活品質等全方位的效果 ，並提供適當可行的飲食方案，以促進糖尿病的良好控制。

（二）研究方法、進行步驟及執行進度。

研究方法

本研究使用隨機對照研究，因為證據等級最高，追蹤 18 個月，因為現有有效證據最多為一年。

第一年：

1. 研究設計：本研究為單一中心，平行設計、隨機對照研究。
2. 研究對象：本研究以 20 歲至 80 歲的第二型糖尿病患者為對象，糖化血色素≧7.5 mg/dl，飲食或藥物控制(降血糖藥、胰島素注射或合併治療)者。但須排除懷孕或哺乳婦女、顯著肝功能異常 (SGOT, SGPT 值≧正常值之三倍)、顯著腎功能異常 (肌酸酐值≧1.5mg/dl，或蛋白尿者)、顯著心臟病(不穩定心絞痛、不穩定心臟衰竭)、頻繁發作之痛風(年發作大於等於三次)、已參加其他減重計畫或使用減重藥物、有精神科飲食疾病(eating disorder)診斷者。能配合兩年研究者。

3. 招募方式(recruitment)：以醫學中心門診、健檢、營養門診、海報招募之糖尿病病人，符合條件者收案，在四個月內完成收案，並實施基本檢查，含所有初級及次級結果的基本測量。

4. 隨機分派方式：研究對象依不同性別、身體質量指數分層(男性與女性；BMI≦24, >24)，以共變量應變式隨機分派 (covariate adaptive randomization)，進入實驗組或對照組。 若同一家族的兩名研究對象進入，則分配在同一組。研究對象在簽妥同意書及基礎評估後，基礎評估包括結果測量之所有項目後，才宣布隨機分配的組別。

5. 介入方式：

(1) 實驗組：營養師低醣飲食衛教(每天醣類攝取≦ 90 克，不限制總熱量) 。為符合醫學倫理，醫師仍須依原則調整藥物，調藥原則：介入組因過去的研究顯示若不調整藥物較容易成低血糖，故一開始就減少硫醯基尿素類口服降血糖藥（Sulfonylurea）以及 Insulin 藥物劑量(調整至一半劑量)。前六個月不加藥，之後每六個月，糖化血色素≧8.0%才增加藥物種類或劑量。期間若任何一次糖化血色素≦6.5%或有因藥物引起低血糖事件發生，才減少藥物種類或劑量。

(2) 對照組: 營養師傳統卡路里限制飲食(calorie-restricted diet)：熱量計算一般人(18.5≦BMI≦24)為每公斤 25 Kcal，肥胖者（BMI>24）使用每公斤 30Kcal，過瘦者(BMI < 18.5)使用每公斤 20Kcal，其中百分比，醣類(carbohydrates)：50-60%，蛋白質(protein)：1.0-1.2 克/公斤 (<20%) and 脂肪≦30%衛教。為符合醫學倫理，醫師仍須依原則調整藥物，調藥原則：前六個月不加藥，之後每六個月，糖化血色素≧8.0%才增加藥物種類或劑量。期間若任何一次糖化血色素≦6.5%或有因藥物引起低血糖事件發生，才減少藥物種類或劑量。

兩組均給予運動建議，同時，並不限制原本已使用的高脂血症用藥、降尿酸藥及 Aspirin 預防性用藥等。兩組分別設 line 群組，分享適合的吃法及外食建議。

訓練時間: 隨機分派後，由營養師根據組別給予營養諮商約 30 分鐘，第二週、第四週、第八週、第十二週給予營養追蹤諮商，回顧飲食及確定飲食控制之正確性。之後每三個月給予營養追蹤諮商，沒有門診的月份，每月由營養師電話追蹤，總計每位個案追蹤 18 個月。營養師並提供食物成分表、料理食譜 、及自我監測建議方式。兩組分別設 line 群組，分享適合的吃法及外食建議。

6. 結果測量：

初級結果(primary outcome)為血糖控制狀況(含飯前、飯後血糖及糖化血色素)、降血糖藥物或胰島素增減種類及劑量，每三個月一次，至第 18 個月。

次級結果(secondary outcome)為：

(1)生化指標: 每三個月測量空腹血脂(含膽固醇、三酸甘油脂、HDL、LDL)、尿酸

(2)內分泌指標：每六個月測量胰島素(胰島素注射者檢驗 c-peptide) 、Apo-B，並計算 HOMA-IR 及 HOMA-beta cell function，至第十八個月

(3)腎功能：第十八個月追蹤微量尿蛋白定量

(4)血壓：每三個月測量收縮壓與舒張壓

(5)身體組成指標：每三個月測量體重、體重質量比、腰圍、臀圍、大腿圍、體脂

(6)功能指標：每三個月測量三公尺走路速度、握力

(7)飲食遵從性：第二、四、八、十二週及之後每三個月的飲食回顧

(8)飲食遵從性的難易度：第二、四、八、十二週及之後每三個月，以李克特量表(Likert scale)測量主觀遵從難易度。

(9)其他代謝性藥物增減狀況：每三個月增減之藥物品項及劑量，含：降血脂藥、降尿酸藥等。

(10) 生活品質：第三個月及第十八個月，評估世界衛生組織生活品質問卷(台灣簡明版)，及糖尿病困擾指數(The 17-item version of the Chinese Diabetes Distress Scale ,CDDS-17)

(11)動脈硬化指標：第十八個月追蹤基礎評估頸動脈或下肢動脈超音波

(12) 眼科眼底檢查：第十八個月追蹤

(13)心血管疾病： 第十八個月心電圖檢查

(14) 癌症：大腸癌(免疫螢光法糞便篩檢)、女性乳癌(乳房攝影)

(15) 脂肪肝及膽結石：第十八個月追蹤腹部超音波

(16)胃食道逆流問卷：胃食道逆流疾病評量問卷(Chinese GERDQ)[78]，第三個月及第十八個月

(17)老人的認知功能及情緒：第十八個月追蹤簡易心智測驗(Mini-State Mental Examination,MMSE), 老年憂鬱量表(Geriatric Depression Scale, GDS-4)

(18)體能活動(physical activity) 第三、六、十二、十八個月追蹤 IPAQ 台灣活動量調查自填短版問卷表(Taiwan International Physical Activity Questionnaire)

(19)老人的認知功能及情緒：第十八個月追蹤簡易心智測驗(Mini-State Mental Examination,MMSE), 老年憂鬱量表(Geriatric Depression Scale, GDS-4)

(20)體能活動(physical activity) 第三、六、十二、十八個月追蹤 IPAQ 台灣活動量調查自填短版問卷表(Taiwan International Physical Activity Questionnaire)

第二年

繼續進行上述有關介入、結果測量及副作用定期監測事項，每位個案完成 18

個月的追蹤，並完成所有資料之輸入。

只有第 18 月的追蹤項目為：

(1) 生活品質：第 18 個月測量世界衛生組織生活品質問卷(台灣簡明版)

(2) 動脈硬化指標：第 18 個月追蹤頸動脈或下肢動脈超音波

(3) 眼科白內障及眼底檢查：第 18 個月追蹤檢查

8. 樣本估計

要能偵測 HbA1c 0.5% (d=0.5)的差別，使用過去文獻[11]之 HbA1c 變化的標準差，估計為 σ = 0.408%，α= 0.05, β=0.2,Zα= 1.96, Zβ= 0.845, P=0.05，假設有 20%的失去追蹤率(p=0.2)，使用公式(來源：臨床試驗樣本數計算簡介 - 財團法人醫藥品查驗中心)如下圖，

$$N = \frac{\left(1+\frac{1}{m}\right)*(Z_\alpha+Z_\beta)^2\sigma^2}{d^2}$$

考慮預期失訪率之樣本數

$$\frac{N}{(1-p)^2}$$

得出所需樣本數一組約為 20 人，但我們選用一組 30 人，比較不會受分佈的影響我們採用分層隨機樣本(stratified random sample)，對初始身體質量指數以及性別來做分層，(BMI≦24, >24、男性與女性)，每層實驗組及對照組兩組，共約 120 人。

9.統計分析

本研究對於初級及次級結果的遺漏值使用最接近的上次結果代替(single imputation method of last measurement carried forward for missing data)，使用本方法的原因是因為可能有高遺漏值，如此可以有較接近的預估值。

本研究使用立意治療分析(intention to treat analysis)。 以 SPSS 17.0 套裝軟體進行統計分析。以平均值±標準差描述基本變項的分佈，以重複測量變異數分析(repeated measures ANOVAs)對於時間變化及各分組變項進行分析。 並依樣本之分佈是否常態，選擇以 independent t tests or Wilcoxon rank tests 比較每次飲食組成之變化。

預計可能遭遇之困難及解決途徑

1.短期需完成收案人數：利用多重管道：門診、健檢、營養門診、海報招募之糖尿病病人

2. 低醣質飲食的接受度：事先強調本研究著重飲食控制，設計方便好用之食譜

3. 病人順從性不佳：每兩週到每月的營養管理師追蹤，兩組分別設 line 群組，分享適合的吃法及外食建議。

進行步驟

第一年

1. 人力招募：遴選具營養背景之研究助理

2. 研究會議：研究團隊召開會議，討論研究細節及流程，確定問卷內容、實施地點及程序。

3. 確定營養諮商內容：文獻回顧、和營養師討論，共同訂定食物成分表、料理食譜 、及自我監測建議方式；並設定每次營養諮商內容及追蹤內容

4.印刷問卷、營養成分表、食譜、追蹤手冊

5.助理訓練：由研究團隊營養師據上述內容，訓練助理，確認能正確執行

6. 收案：門診、健檢、營養門診、海報招募之糖尿病病人，符合條件者轉介，經初評及解說，能配合兩年研究者收案。

7. 隨機分配進入實驗組或對照組。

8. 介入計畫：各依實驗組及對照組介入方式進行介入及追蹤

9. 結果測量: 依照訂定之初級指標及次級指標收集資料

10.副作用監測：依照所訂定之監測方式收集資料

11.資料收集及輸入：將上述結果輸入電腦

第二年

1. 介入計畫：各依實驗組及對照組介入方式進行介入及追蹤
2. 結果測量: 依照訂定之初級指標及次級指標收集資料
3. 副作用監測：依照所訂定之監測方式收集資料
4. 資料收集及輸入：將上述結果輸入電腦
5. 資料分析：使用套裝統計軟體進行分析
6. 報告撰寫

執行進度

月份	2	4	6	8	10	12	14	16	18	20	22	24
人力招募	—											
研究會議	—	—										
確定營養諮商內容	—	—										
印刷問卷、營養成分表、食譜、追蹤手冊	—	—										
助理訓練	—	—										
收案		—	—									
隨機分配		—	—									

介入計畫												
結果測量												
副作用監測												
資料收集及輸入												
資料分析												
報告撰寫												

（三） 預期完成之工作項目、成果及績效。

1.預期完成之工作項目

第一年：

(1) 各類飲食之含醣成分估計及代換表
(2) 低醣飲食之內容
(3) 低醣飲食之食譜
(4) 低醣飲食之外食建議
(5) 低醣飲食之自我估計表
(6) 完成收案、基本評估及隨機分組
(7) 使用 line 群組增進飲食控制的內容
(8) 每個月個案飲食遵從度
(9) 每三個月個案完成追蹤率
(10) 完成每三個月定期追蹤項目
(11) 完成每三個月副作用監測

第二年：

(1)每個月個案飲食遵從度至第十八個月
(2) 每三個月個案完成追蹤率至第十八個月
(3) 完成每三個月定期追蹤項目至第十八個月
(4) 完成每三個月副作用監測至第十八個月
(5) 完成資料輸入及除錯
(6) 完成統計分析
(7) 完成報告撰寫

2.對於學術研究、國家發展及其他應用方面預期之貢獻

第一年:

(1) 本國首次低醣飲食的研究
(2) 低醣飲食對糖尿病控制之短期成效
(3) 低醣飲食對糖尿病生活品質提升的短期影響
(4) 低糖飲食的副作用監測建立

第二年:

(1)　低醣飲食對糖尿病控制之長期成效
(2)　低醣飲食對糖尿病生活品質提升的長期影響
(3)　可基於此有效之結果,再擴大樣本數，進行後續心血管疾病及死亡率之分析

3.對於參與之工作人員，預期可獲之訓練。
第一年
(1)　低醣飲食的教育模式建立
(2)　低醣飲食的食譜建立
(3)　低醣飲食的外食建議建立
(4)　增進低醣飲食遵從度的方法

第二年
(1)　低醣飲食對於糖尿病藥物減少的幅度
(2)　低醣飲食對於糖尿病控制的成效
(3)　低醣飲食的滿意度

4. 預期完成之研究成果及績效（如期刊論文、研討會論文、專書、技術報告、專利或技術移轉等質與量之預期績效）
第一年
低醣飲食研討會論文
低醣飲食期刊論文
第二年
低醣飲食研討會論文
低醣飲食期刊論文
低醣飲食專書

参考文獻

1. Ajala O, English P, Pinkney J.:Systematic review and meta-analysis of different dietary approaches to the management of type 2 diabetes. Am J Clin Nutr. 2013 Mar;97(3):505-16.
2. Standards of Medical Care in Diabetes 2007. Diabetic Care. 2007;30(supp1): S4-38.
3. Standards of Medical Care in Diabetes 2008. Diabetic Care. 2008;31(supp1): S12-54.
4. Standards of Medical Care in Diabetes 2011. Diabetic Care. 2011;34(supp1): S11-51.
5. Life with Diabetes: A Series of Teaching Outlines Paperback – November 19, 2009 By The Michigan Diabetes Research and Training Center.
6. Standards of Medical Care in Diabetes 2014. Diabetic Care. 2013;36(supp1): S12-56.
7. Standards of Medical Care in Diabetes 2014. Diabetic Care. 2014;37(supp1): S14-80.
8. Feinman RD, Pogozelski WK, Astrup A et al: Dietary carbohydrate restriction as the first approach in diabetes management: Critical review and evidence base. Nutrition. 2014 Jul 16.
9. Samaha FF, Iqbal N, Seshadri P, Chicano KL, Daily DA, McGrory J,Williams T, Williams M, Gracely EJ, Stern L. A low-carbohydrate as compared with a low-fat diet in severe obesity. N Engl J Med 2003;348:2074–81.
10. Stern L, Iqbal N, Seshadri P, Chicano KL, Daily DA, McGrory J,Williams M, Gracely EJ, Samaha FFThe effects of low-carbohydrate versus conventional weight loss diets in severely obese adults: one year follow-up of a randomized trial. Ann Intern Med 2004;140:778–85.
11. Westman EC, Yancy WS, Mavropoulos JC, Marquart M, McDuffie JR.:The effect of a low-carbohydrate, ketogenic diet versus a lowglycemic index diet on glycemic index diet on glycemic control in type 2 diabetes mellitus. Nutr Metab (Lond) 2008;5:36.
12. Wolever TM, Gibbs AL, Mehling C, Chiasson JL, Connelly PW, Josse RG, Leiter LA, Maheux P, Rabasa-Lhoret R, Rodger NW, et al. The Canadian Trial of Carbohydrates in Diabetes (CCD), a 1-y controlled trial of low-glycemic-index dietary carbohydrate in type 2 diabetes: no effect on glycated hemoglobin but reduction in C-reactive protein. Am J Clin Nutr 2008;87:114–25.
13. Haimoto H, Iwata M,Wakai K, Umegaki H. Long-term effects of a diet loosely restricting carbohydrates on HbA1c levels, BMI and tapering of sulfonylureas in type 2 diabetes: a 2-year follow-up study. Diabetes Res Clin Pract 2008 ;79:350–6.
14. Davis NJ, Tomuta N, Schechter C, Isasi CR, Segal-Isaacson CJ, Stein D, Zonszein J, Wylie-Rosett J. Comparative study of the effects of a 1-year dietary intervention of a low-carbohydrate diet versus a low- fatdiet on weight and glycemic control in type 2 diabetes. Diabetes Care 2009;32:1147–52.
15. Elhayany A, Lustman A, Abel R, Attal-Singer J, Vinker S. A low carbohydrate Mediterranean diet improves cardiovascular risk factors and diabetes control among overweight patients with type 2 diabetes mellitus: a 1-year prospective randomized intervention study. Diabetes Obes Metab 2010;12:204–9.

16. Yang WS Jr, Westman EC, McDuffie JR, Grambow SC, Jeffreys AS, Bolton J, Chalecki A, Oddone EZ. A randomized trial of a lowcarbohydrate diet vs orlistat plus a low-fat diet for weight loss. Arch Intern Med 2010;170:136–45.
17. Iqbal N, Vetter ML, Moore RH, Chittams JL, Dalton-Bakes CV, Dowd M, Williams-Smith C, Cardillo S, Wadden TA. Effects of a low-intensity intervention that prescribed a low-carbohydrate vs. a low-fat diet in obese. Obesity (Silver Spring) 2010;18:1733–8.
18. Yamada Y, Uchida J, Izumi H, Tsukamoto Y, Inoue G, Watanabe Y, Irie J, Yamada S:.A non-calorie-restricted low-carbohydrate diet is effective as an alternative therapy for patients with type 2 diabetes. Intern Med. 2014;53(1):13-9.
19. Tirosh A, Golan R, Harman-Boehm I, Henkin Y, Schwarzfuchs D, Rudich A, 20.
Kovsan J, Fiedler GM, Bl"uher M, Stumvoll M, Thiery J, Stampfer MJ, Shai I:Renal function following three distinct weight loss dietary strategies during 2 years of a randomized controlled trial. Diabetes Care. 2013 Aug;36(8):2225-32
20. Santos FL, Esteves SS, da Costa Pereira A, Yancy WS Jr, Nunes JP. Systematic review and meta-analysis of clinical trials of the effects of low carbohydrate diets on cardiovascular risk factors. Obes Rev. 2012 Nov;13(11):1048-66.
21. Ruth MR, Port AM, Shah M, Bourland AC, Istfan NW, Nelson KP, Gokce N, Apovian CM: Consuming a hypocaloric high fat low carbohydrate diet for 12 weeks lowers C-reactive protein, and raises serum adiponectin and high density lipoprotein -cholesterol in obese subjects. Metablosim 2013 Dec;62(12):1779-87.
22. Larsen RN, Mann NJ, Maclean E, Shaw JE. The effect of high-protein, low-carbohydrate diets in the treatment of type 2 diabetes: a 12 month randomised controlled trial Diabetologia. 2011;54(4):731-40.
23. Guldbrand H1, Dizdar B, Bunjaku B, Lindström T, Bachrach-Lindström M, Fredrikson M, Ostgren CJ, Nystrom FH: In type 2 diabetes, randomisation to advice to follow a low-carbohydrate diet transiently improves glycaemic control compared with advice to follow a low-fat diet producing a similar weight loss. Diabetologia. 2012 Aug;55(8):2118-27.
24. Krebs JD1, Elley CR, Parry-Strong A, Lunt H, Drury PL, Bell DA, Robinson E, Moyes SA, Mann JI: The Diabetes Excess Weight Loss (DEWL) Trial: a randomised controlled trial of high-protein versus high-carbohydrate diets over 2 years in type 2 diabetes. Diabetologia. 2012 Apr;55(4):905-14.
25. Dyson PA, Beatty S, Matthews DR An assessment of low-carbohydrate or low-fat diets for weight loss at 2 year's follow-up. Diabet Med. 2010 Mar;27(3):363-4.
26. Hussain TA, Mathew TC, Dashti AA, Asfar S, Al-Zaid N, Dashti HM. Effect of low-calorie versus low-carbohydrate ketogenic diet in type 2 diabetes. Nutrition. 2012 Oct;28(10):1016-21.
27. Centers for Disease Control and Prevention (CDC). Trends in intake of energy and macronutrients--United States, 1971-2000. MMWR Morb Mortal Wkly Rep. 2004 Feb 6;53(4):80-2.
28. J.S. Volek, M.L. Fernandez, R.D. Feinman, S.D. Phinney Dietary carbohydrate restriction induces a unique metabolic state positively affecting atherogenic dyslipidemia, fatty acid partitioning, and metabolic syndrome. Prog Lipid Res, 47 (2008), pp. 307–318.

29. A. Al-Khalifa, T.C. Mathew, N.S. Al-Zaid, E. Mathew, H.M. Dashti Therapeutic role of low-carbohydrate ketogenic diet in diabetes. Nutrition, 25 (2009), pp. 1177–1185.
30. M.C. Gannon, H. Hoover, F.Q. Nuttall Further decrease in glycated hemoglobin following ingestion of a LoBAG30 diet for 10 wk compared with 5 wk in people with untreated type 2 diabetes. Nutr Metab (Lond), 7 (2010), p. 64.
31. P.A. Dyson, S. Beatty, D.R. Matthews A low-carbohydrate diet is more effective in reducing body weight than healthy eating in both diabetic and non-diabetic subjects. Diabet Med, 24 (2007), pp. 1430–1435.
32. B.V. Howard, J.E. Manson, M.L. Stefanick, S.A. Beresford, G. Frank, B. Jones, et al. Low-fat dietary pattern and weight change over 7 y: the Women’s Health Initiative dietary modification trial. JAMA, 295 (2006), pp. 39–49.
33. R.C. Atkins. Dr. Atkins’ new diet revolution. Avon Books, New York (2002)
34. E.C. Westman, S.D. Phinney, J. Volek The new Atkins for a new you: the ultimate diet for shedding weight and feeling great forever. Simon & Schuster, New York (2010).
35. Gunnars, K. Low-Carb Diets – Healthy, but Hard to Stick to? In: Authority Nutrition. 2013. Available at http://AuthorityNutrition.com. Accessed September 6, 2014.
36. A. Belza, C. Ritz, M.Q. Sorensen, J.J. Holst, J.F. Rehfeld, A. Astrup Contribution of gastroenteropancreatic appetite hormones to protein-induced satiety. Am J Clin Nutr, 97 (2013), pp. 980–989.
37. R.D. Feinman, M.C. Vernon, E.C. Westman Low carbohydrate diets in family practice: what can we learn from an internet-based support group. Nutr J, 5 (2006), p. 26
38. J.W. Krieger, H.S. Sitren, M.J. Daniels, B. Langkamp-Henken Effects of variation in protein and carbohydrate intake on body mass and composition during energy restriction: a meta-regression 1. Am J Clin Nutr, 83 (2006), pp. 260–274.
39. R.D. Feinman, E.J. Fine “A calorie is a calorie” violates the second law of thermodynamics. Nutr J, 3 (2004), p. 9
40. R.D. Feinman, E.J. Fine Nonequilibrium thermodynamics and energy efficiency in weight loss diets. Theor Biol Med Model, 4 (2007), p. 27
41. Siri-Tarino, P.W., Sun, Q., Hu, F.B., and Krauss, R.M. Meta-analysis of prospective cohort studies evaluating the association of saturated fat with cardiovascular disease. Am J Clin Nutr. 2010; 91: 535–546.
42. Siri-Tarino, P.W., Sun, Q., Hu, F.B., and Krauss, R.M. Saturated fat, carbohydrate, and cardiovascular disease. Am J Clin Nutr. 2010; 91: 502–509
43. Weinberg, S.L. The diet-heart hypothesis: a critique. J Am Coll Cardiol. 2004; 43: 731–733.
44. Ravnskov, U., Rosch, P.J., Sutter, M.C., and Houston, M.C. Should we lower cholesterol as much as possible?. Bmj. 2006; 332: 1330–1332.
45. Yancy, W.S. Jr., Westman, E.C., French, P.A., and Califf, R.M. Diets and clinical coronary events: The truth is out there. Circulation. 2003; 107: 10–16
46. Teicholz, N. The big fat surprise. Why butter, meat & cheese belong in a healthy diet. Simon & Schuster, New York; 2014.
47. Kendrick, M. The great cholesterol con: the truth about what really causes heart disease and how to avoid it. John Blake, London; 2008.

48. Ravnskov, U. The cholesterol myths: exposing the fallacy that cholesterol and saturated fat cause heart disease. NewTrends Publishing, Inc., Washington, DC; 2000.
49. Taubes, G. Good calories, bad calories. Alfred A. Knopf, New York; 2007.
50. Jakobsen, M.U., O'Reilly, E.J., Heitmann, B.L., Pereira, M.A., Balter, K., Fraser, G.E. et al. Major types of dietary fat and risk of coronary heart disease: a pooled analysis of 11 cohort studies. Am J Clin Nutr. 2009; 89: 1425–1432.
51. Lin, J., Wu, P.H., Tarr, P.T., Lindenberg, K.S., St-Pierre, J., Zhang, C.Y. et al. Defects in adaptive energy metabolism with CNS-linked hyperactivity in PGC-1 alpha null mice. Cell. 2004; 119: 121–135.
52. Forsythe, C.E., Phinney, S.D., Feinman, R.D., Volk, B.M., Freidenreich, D., Quann, E. et al. Limited effect of dietary saturated fat on plasma saturated fat in the context of a low carbohydrate diet. Lipids. 2010; 45: 947–962.
53. Forsythe, C.E., Phinney, S.D., Fernandez, M.L., Quann, E.E., Wood, R.J., and Bibus, D.M. Comparison of low fat and low carbohydrate diets on circulating fatty acid composition and markers of inflammation. Lipids. 2008; 43: 65–77.
54. Volek, J.S., Phinney, S.D., Forsythe, C.E., Quann, E.E., Wood, R.J., Puglisi, M.J. et al. Carbohydrate restriction has a more favorable impact on the metabolic syndrome than a low fat diet. Lipids. 2009; 44: 297–309.
55. Stratton, I.M., Adler, A.I., Neil, H.A., Matthews, D.R., Manley, S.E., Cull, C.A. et al. Association of glycaemia with macrovascular and microvascular complications of type 2 diabetes (UKPDS 35): prospective observational study. BMJ. 2000; 321: 405–412.
56. Turner, R.C. The U.K. Prospective Diabetes Study. A review. Diabetes Care. 1998; 21: C35–38.
57. Turner, R.C., Millns, H., Neil, H.A., Stratton, I.M., Manley, S.E., Matthews, D.R. et al. Risk factors for coronary artery disease in non-insulin dependent diabetes mellitus: United Kingdom Prospective Diabetes Study (UKPDS: 23). BMJ. 1998; 316: 823–828.
58. Jenkins, D.J., Kendall, C.W., McKeown-Eyssen, G., Josse, R.G., Silverberg, J., Booth, G.L. et al. Effect of a low-glycemic index or a high-cereal fiber diet on type 2 diabetes: a randomized trial. JAMA. 2008; 300: 2742–2753.
59. Feinman, R.D., Volek, J.S., and Westman, E. Dietary carbohydrate restriction in the treatment of diabetes and Mmtabolic syndrome. Clin Nutr Insight. 2008; 34: 1–5.
60. Barter, P.J., Ballantyne, C.M., Carmena, R., Castro Cabezas, M., Chapman, M.J., Couture, P. et al. Apo B versus cholesterol in estimating cardiovascular risk and in guiding therapy: Report of the thirty-person/ten-country panel. J Intern Med. 2006; 259: 247–258.
61. Dreon, D.M., Fernstrom, H.A., Campos, H., Blanche, P., Williams, P.T., and Krauss, R.M. Change in dietary saturated fat intake is correlated with change in mass of large low-density-lipoprotein particles in men. Am J Clin Nutr. 1998; 67: 828–836.
62. Dreon, D.M., Fernstrom, H.A., Williams, P.T., and Krauss, R.M. A very low-fat diet is not associated with improved lipoprotein profiles in men with a predominance of large, low-density lipoproteins. Am J Clin Nutr. 1999; 69: 411–418.

63. McLaughlin, T., Reaven, G., Abbasi, F., Lamendola, C., Saad, M., Waters, D. et al. Is there a simple way to identify insulin-resistant individuals at increased risk of cardiovascular disease?. Am J Cardiol. 2005; 96: 399–404.
64. Nielsen, J.V., Gando, C., Joensson, E., and Paulsson, C. Low carbohydrate diet in type 1 diabetes, long-term improvement and adherence: a clinical audit. Diabetol Metab Syndr. 2012; 4: 23.
65. Bernstein, R.K. Dr. Bernstein's diabetes solution: the complete guide to achieving normal blood sugars. 4th ed. Little, Brown and Co, New York; 2011.
66. Saslow, L.R., Kim, S., Daubenmier, J.J., Moskowitz, J.T., Phinney, S.D., Goldman, V. et al. A randomized pilot trial of a moderate carbohydrate diet compared with a very low carbohydrate diet in overweight or obese individuals with type 2 diabetes mellitus or prediabetes. PLoS One. 2014; 9: e91027.
67. Yancy, W.S. Jr., Foy, M., Chalecki, A.M., Vernon, M.C., and Westman, E.C. A low-carbohydrate, ketogenic diet to treat type 2 diabetes. Nutr Metab (Lond). 2005; 2: 34.
68. Nielsen, J.V. and Joensson, E. Low-carbohydrate diet in type 2 diabetes. Stable improvement of bodyweight and glycemic control during 22 mo follow-up. Nutr Metab (Lond). 2006; 3: 22.
69. Nielsen, J.V., Jonsson, E., and Nilsson, A.K. Lasting improvement of hyperglycaemia and bodyweight: low-carbohydrate diet in type 2 diabetes—a brief report. Ups J Med Sci. 2005; 110: 69–73.
70. Boden, G., Sargrad, K., Homko, C., Mozzoli, M., and Stein, T.P. Effect of a low-carbohydrate diet on appetite, blood glucose levels, and insulin resistance in obese patients with type 2 diabetes. Ann Intern Med. 2005; 142: 403–411.
71. Gerstein, H.C., Miller, M.E., Byington, R.P., Goff, D.C. Jr., Bigger, J.T., Buse, J.B. et al. Effects of intensive glucose lowering in type 2 diabetes. N Engl J Med. 2008; 358: 2545–2559.
72. 江部 康二 糖尿病治療のための! 糖質制限食パーフェクトガイド 2013/8/9.
73. 江部 康二 主食を抜けば糖尿病は良くなる! 新版: 糖質制限食のすすめ 2014/3/14.
