# Supplementary material for: Effect of a 90 g/day low-carbohydrate diet on glycaemic control, small, dense low-density lipoprotein and carotid intima-media thickness in type 2 diabetic patients: An 18-month randomised controlled trial
Source: PLoS One. 2020 Oct 5;15(10):e0240158. doi: 10.1371/journal.pone.0240158 (PMC7535044; doi:10.1371/journal.pone.0240158)
Supplement: S2 File — (PDF) [file pone.0240158.s003.pdf]

The Effectiveness of Low Carbohydrate Diet in Reducing Polypharmacy for Patients with Type 2 Diabetes Mellitus

A. English Abstract of Project

Most of the Diabetic Associations recommend a carbohydrate intake of 50-60% of total energy intake, total fat intake ≦30% of energy. However, there is insufficient evidence, including systematic review or meta-analysis, to justify these recommendations. More and more evidence supports the effect and convenience of low carbohydrate diet, but the long-term evidence is insufficient. The lack of evidence could be due to inadequate carbohydrate restriction, non-compliance or the adjustment of hypoglycemic agents mask the real effect of diet control.

Our randomized controlled trial is designed for age 20 to 80 type II Diabetes Mellitus outpatients with HbA1C≧7.5. They will be stratified according to sex and body mass index(BMI) (BMI≦24, >24, man or woman).

The sample size is estimated to be 120 in total. The experimental group is low carbohydrate diet group (carbohydrate less than 90g/day without limitation of caloric intake). The control group is traditional caolorie-restricted diet: carbohydrate 50-60%, protein1.0-1.2 g/kg (<20%) and fat≦30%). The hypoglycemic agents are adjusted by the physicians according to the study rule. The follow-up time is 18 months. The primary outcome is the adjustment of medication (types or dosage), the scores of medication effect score calculation and glycemic control (HbA1c, fasting glucose and 2hr glucose).

The secondary outcomes are biochemical change, metabolic change, physical assessment, functional assessment, atherosclerosis assessment and Diabetes Distress Scale (The 17-item version of the Chinese Diabetes Distress Scale,CDDS-17) . Our aim is to test the effectiveness of low carbohydrate diet to improve the diabetic control and reduce the polypharmacy.

B. Content of Project

(A) Background and purpose

Diabetes is a major risk factor for cardiovascular disease, and comorbidities such as hypertension, hyperlipidemia, ischemic heart disease, heart failure, cerebrovascular disease, peripheral vascular disease results in dementia, depression, atherosclerosis and frailty. Excluding cancer-related frailty, vascular frailty should return to the prevention and treatment of major chronic diseases such as diabetes in order to prevent aging. Therefore, the best treatment of diabetes is an important preventive indicator for an aging society.

The diet recommendations for type 2 diabetes are similar between different associations, such as: British Diabetes Association, European Association for the Study of Diabetes (EASD), American Diabetes Association (ADA), American Heart Association, Canadian Diabetes Association, International College of Nutrition, Groups from South Africa and Japan, and the National Cholesterol Education Panel (Adult Treatment Panel 3). The traditionally diabetic diet recommended 50-60% of intake from carbohydrates and less than 30% from fat. But there is not enough medical evidence, including: a systematic review or meta-analysis to support such a proposal [1].

The 2007ADA “Standards of Medical Care in Diabetes” did not recommend low-carb diets because there was not enough evidence to support the long-term effects and the risk of cardiovascular disease was still unclear [2]. But in 2008 ADA changed the discourse [3], they suggested short-term use of low-fat diet or a low carbohydrate diet for overweight diabetics or non-diabetics.(for one year in 2008, and 2 years in 2011 [4].

In fact, ADA published “Life With Diabetes” with documentation of "Carbohydrate, protein and lipid provide calories, but only carbohydrate affects blood glucose " [5] . In 2013, the ADA even removed the words that 130 grams of carbohydrates a day was required [6]. In 2014, ADA mentioned low fat diet can’t achieve good blood glucose control and reduce the cardiovascular risk by meta-analysis and systematic review [7]. In 2014, Standards of Medical Care in Diabetes provides several dietary methods, including 1. Mediterranean diet 2. Vegetarian and vegan diet 3. Low fat diet 4. Low carbohydrates diet 5. DASH (Dietary Approaches to Stop Hypertension). The ADA was not able to come up with the most ideal diet, and it was the same in 2015

Although the low-carbohydrate diet is not mainstream, there is growing evidence that such treatments are low-risk and easy to follow [2] . The low carbohydrate diet, in accordance with the latest proposal [8]: classified as: (1) very low-carbohydrate ketogenic diet, VLCKD, the intake of carbohydrate per day of 20 -50 grams, or less than 10% of a daily diet of 2000 calories; (2) low- carbohydrate diet : the intake of carbohydrates is less than 130 grams per day , or less than 26% of total daily energy ; (3) medium carbohydrate diet : the intake of carbohydrate is 26-45% of the total daily energy per day ; (4) High-carbohydrate diet : The intake of glycogen is > 45% of the total daily energy of the daily diet.

A recent systematic review [1] found that low carbohydrate diet, low glycemic index diet, Mediterranean diet and high protein diet control blood glucose better than the control group. Low-carbohydrate diet and Mediterranean diet were most effective for weight loss.

Most of the review literatures [9-17] targeted on obese persons [9-11, 14-17] , three articles included 30-40% of diabetic patients [9.10.16] and four focused on obese diabetic patients [11 , 14, 15, 17] , only two are not for obese persons, including a study for diet-controlled diabetic patient [12] and a study for general diabetes patients [13] . In the study of mixed diabetic and non-diabetic patients, the 6 -month study [9] showed significant improvement in body weight, blood glucose, and triglyceride; however, in the one- year study [10], low carbohydrate diet showed no significant weight loss effect, possible due to good diet control in control group, poor compliance of low carbohydrate diet , high drop out rate. The other one-year study compared low-carb diets and low-fat diets with weight loss pills “Orlistat “[16].There was no significant difference between the two groups. The low carbohydrate intake at 48 weeks for this study was 62 Gram / day (15% calories) , showing good compliance.

In a 6-month study of patients with diabetes, 40% carbohydrate diet was not superior to traditional diabetic diet on glycemic control [12]. In another non-randomized controlled study in Japan for two years, 40% of carbohydrates showed, a better control on blood glucose, weight, total cholesterol and low density lipoprotein, reducing the types and dosage of oral hypoglycemic agents, especially sulfonylurea [13] . In a study for overweight diabetic patients, very low carbohydrate diet (20-30 grams/day) showed effectiveness in reduction of HbA1c and body weight in the first 3 months. But it turned to be 77 ± 44 g carbohydrate/day (24% of calories) after one

year and showed no difference between low carbohydrate diet and control groups [14].

A recent randomized controlled trial of 70-130 g carbohydrate / day (n=24) in Japan demonstrated that type 2 diabetes with recent HbA1c 6.9 to 8.4% reduced HbA1c 0.5% at 6 months (P = 0.03) [18] .

For renal function, a randomized controlled trial of three different diets (low carbohydrate diet, Mediterranean diet, low-fat diet) published in Diabetes Care [19] in August 2013 (n=318) , showed that all of the above diets were beneficial in weight loss and increased eGFR in chronic kidney disease(CKD) stage III or less patients in two years and without difference between groups.

For cardiovascular risk factors, such as lipid profile and CRP, a systematic review of low carbohydrate diets published in the Obes Rev in November 2012 [20], showed significant reduction of body weight, body mass index (BMI), blood pressure, Abdominal circumference , triglyceride (TG), HbA1c, insulin, and CRP . There was significant increase of HDL and no significant change of LDL and creatinine. Another study showed that a low carbohydrate diet reduced hs-CRP and increase total Adiponectin in three months [21].

Long-term randomized controlled trials revealed that high-protein low-carbohydrate diets didn’t show significant advantage in HbA1c and lipid metabolism than high-carbohydrate diets for patients with type 2 overweight or obese diabetes in one year follow-up [22]. However, the high protein low-carbohydrate diet was 30% protein and 40% carbohydrate, compared with 15% protein and 55% carbohydrate in high carbohydrate diet. There may be behavioral changes in both groups, or similar self-reported dietary status and food choices. The sample size is 93 with adequate power. However, the carbohydrate intake was not shown in the study. A two-year research based on the same low-carbohydrate formula (n=419)[23] (200g carbohydrate/day in a daily energy of 2000 kcal) didn’t show benefit than the traditional diabetic diet. It is possible that more carbohydrate restriction result in better control. Another two-year study in overweight or obese type 2 diabetes restricted 20% daily carbohydrate in men and women (total energy1800 kcal and 1600 kcal/day respectively) with small sample size 61. There is no significant difference in body weight, HbA1c , insulin and high-density lipoprotein.

In a small sample size study with half diabetes and non-diabetes (n=26) [25], low carbohydrate diet with 40 grams carbohydrate per day showed significant reduction in

HbA1c and body weight in three months, but no significant benefit after two years of observation .

Literature 8 made a systematic review for low carbohydrate diet and proposed the 12 points of evidences:

1. Hyperglycemia is the most salient feature of diabetes. Dietary carbohydrate restriction has the greatest effect on decreasing blood glucose levels. In a prospective study [14]compared a VLCKD with a low-calorie diet over a 24-wk period in 102 diabetic and 261 nondiabetic individuals , blood glucose dropped more dramatically in the VLCKD group than in those given the low-calorie diet (HbA1c: VLCKD: 6.2%, low-calorie diet: > 7.5%).
2. During the epidemics of obesity and type 2 diabetes, caloric increases have been due almost entirely to increased carbohydrates. Data from the National Health and Nutrition Examination Surveys (NHANES) indicate a large increase in carbohydrates as the major contributor to caloric excess in the United States from 1974 to 2000. From the time of the first NHANES study (1974) to the last (2000), dietary carbohydrate in men rose from 42% to 49% of calories. For women, carbohydrate rose from 45% to 52%. The absolute amount of fat decreased for men during this period and showed only a slight increase for women. Data reveals the rise, during this period, of the incidence of type 2 diabetes to its current near epidemic proportions [27] Studies have shown that [28] the stimulation of insulin secretion increases the anabolism of triglycerides (TG) and other TG-rich lipoproteins. It is also believed that fat accumulation in the liver and pancreas will increase the production of VLDL and bring fat to the pancreas, which will have a negative effect on the function of beta-cell [29].
3. Benefits of dietary carbohydrate restriction do not require weight loss. Type 2diabetes took limited carbohydrate diet (30% carbohydrate (CHO), 30% protein, 40% fat, (30:30:40) , which didnnnot fulfill the low carbohydrate diet definition (<130g/d or <26% total energy) , got better glycemic control despite no significant weight loss after ten weeks [30].
4. Although weight loss is not required for benefit, no dietary intervention is better than carbohydrate restriction for weight loss. One study [31] randomly allocated 26 people to either a low-carbohydrate diet (40 g/d carbohydrate) or a “healthy-eating diet” following Diabetes UK nutritional recommendations for 3 mo. Thirteen people with type 2 diabetes and 13 controls without diabetes were included. Weight loss was greater in the low-carbohydrate arm (6.9 versus 2.1 kg). The Women's Health Initiative (WHI) is the most recent example. In the study [32], diet performance in 48,000 postmenopausal women was compared with

usual behavior. The low-fat intervention group had modest weight loss (average 2.2 kg) occurred in the first year, but regained weight at the end of study. The low-carbohydrate diets like the Atkins diet [33,34] put no formal limit on caloric consumption on the assumption that the greater satiety of protein and fat will provide control of intake.

5. Adherence to low-carbohydrate diets in people with type 2 diabetes is at least as good as adherence to any other dietary interventions and is frequently significantly better [35-37]. The good adherence is because of easy to follow without calculation of energy, greater satiety of protein and fat, decreasing fluctuation of blood glucose, reducing the diabetic medication and insulin use.
6. Replacement of carbohydrate with protein is generally beneficial effect on weight loss, body composition, resting metabolic rate, and cardiovascular risk than fat-reduced diets [38-40].
7. Dietary total and saturated fat do not correlate with risk for cardiovascular disease.
8. Plasma saturated fatty acids are controlled by dietary carbohydrate more than by dietary lipids It is increasingly understood that plasma SFAs are associated with increased risk for CVD and insulin resistance [51] . In humans, plasma SFAs do not correlate with dietary saturated fat but, rather, are more dependent on dietary carbohydrates [52-54]. Elevated SFAs arise from increased production of TG-containing lipoproteins, reduced clearance, and the effect of dietary carbohydrate on de novo fatty acid synthesis.
9. The best predictor of microvascular and, to a lesser extent, macrovascular complications in patients with type 2 diabetes, is glycemic control (HbA1c) There was a 14% decrease in MI for every 1% reduction in HbA1c. There was a dramatic 37% decrease in these end points for microvascular risk for each 1% reduction in HbA1c[55-57].
10. Dietary carbohydrate restriction is the most effective method (other than starvation) of reducing serum TGs and increasing high-density lipoprotein. The low-GI diet increases the high-density lipoprotein (HDL) levels [11, 58-59]. Total and/or LDL cholesterol are the most commonly assessed lipid markers for CVD risk despite the general recognition that they are not good predictors[60-62]. Several other parameters have been shown to provide stronger evidence of risk and these tend to be reliably improved by dietary carbohydrate restriction. These include apolipoprotein (apo) B , ratio of total cholesterol to HDL, higher populations of the smaller dense LDL known as pattern B , as well as the ratio of apoB to apoA1. The ratio of TG to HDL, which is also improved more by carbohydrate restriction is taken as a correlate of the smaller dense LDL, which is not routinely measured [63].

11. Patients with type 2 diabetes on carbohydrate-restricted diets reduce and frequently eliminate medication. People with type 1 usually require lower insulin [64-70].
12. Intensive glucose lowering by dietary carbohydrate restriction has no side effects comparable to the effects of intensive pharmacologic treatment.The ACCORD (Action to Control Cardiovascular Disease in Diabetes) trial revealed the intensive-therapy group with more cardiovascular mortality and hypoglycemia compared with the standard-therapy group [71]. The low carbohydrate diet improves the glycemic control, reduces medications and decreases cost of pharmacy.

In Japan, Dr. Ebe at Takao Hospital in Kyoto finds the effectiveness of low carbohydrate diet after he has diabetes , does research on the nature of diabetes, and treats his patients with this special diet [72-73]. He promotes the low-carbohydrate diet to the Japanese medical system and society. He is well recognized and becomes a bestseller in Japan. Japanese restaurants follow to design low-carb menu.

In summary, the low-carbohydrate diet is gradually accepted in the guidelines of the ADA, and is also considered as the initial treatment for diabetes, but there is still no evidence of long-term (more than one year) effectiveness; the study is mostly limited to overweight or obese diabetic patients without data for the normal or low BMI diabetic patients in the real world; the best low carbohydrate restriction to take into account the effectiveness and durability is inconclusive; it is still lack of long-term effectiveness; the reduction of drug requirement should be taken into consideration of the effectiveness. For the long term diabetic control, too many drugs increase medical expenses and expose secondary drug failure risk. The easy principle of low carbohydrate diet is worthy to have further study to confirm the glycemic control in long-term.

In order to determine the long-term effect and safety of LCD on type 2 DM with a reasonable, effective and tolerable carbohydrate intake, a clinical trial will be designed and conducted for the effectiveness of glycemic control, reduction of polypharmacy, biochemical markers, body composition measurement, metabolic markers , functional assessment, atherosclerosis and quality of life.

(B) Methods and flowchart

Methods:

Type of study: a randomized controlled trial for 18 months. (Current evidence is

12-month)

First year:

1. Study design: a single-centered, parallel-designed, randomized controlled trial
2. Study population: Adults, 20–80 years of age, with type 2 DM will be recruited if they are diagnosed with diabetes ≥1 year ago, regardless of whether they receive treatment [oral hypoglycemic agents (OHA) and/or insulin treatment], and if they have a poorly controlled HbA1c ≥ 58 mmol/mol (7.5%) in the previous 3 months. The patients will be identified for inclusion by physicians' referrals from outpatient clinics at a medical center and will be verified by a research assistant to conduct the screening. The exclusion criteria includes pregnant or lactating women, impaired renal function with a serum creatinine ≥ 132.6μmol/L (1.5 mg/dL), abnormal liver function (alanine aminotransferase (ALT), aspartate aminotransferase ≥3 times the normal upper limit) or liver cirrhosis, significant heart diseases (unstable angina, unstable heart failure), frequent gout attacks (≥3 times/year), participation in other weight-loss programs or the use of weight-loss drugs, eating disorders, and inability to complete the questionnaire .
3. Recruitment: The patients will be referred from outpatient clinics, health check-up, dietitian clinics, and poster announcement. We expect to recruit the patients within 4 months and do the baseline assessment.
4. Randomization: The study population will be allocated sequence implementation using Taves covariate-adaptive randomization, stratified using sex and BMI (<24 and ≥24).If the members of the same family are recruited, they will be assigned in the same group. The study population will be assigned to the study group after they complete the baseline assessment.
5. Intervention:

For the LCD group, the daily carbohydrate intake will be limited to less than 90 g, without any restriction to total energy. For those with good dietary compliance, sulfonylurea and insulin injections will be reduced to half doses in advance to prevent hypoglycemia.

For the CRD group, the target total calorie intake will be calculated by multiplying the ideal body weight by 25 kcal/kg for those with a BMI between 18.5–24, 20 kcal/kg for obese subjects with a BMI >24 and 30 kcal/d for underweight subjects with a BMI <18.5. The macronutrient percentage will be 50–60% for carbohydrates, 1.0–1.2 g/kg for protein, and for fat it was ≤30%.

The medication for both groups will be adjusted every 6 months if HbA1c is more than 64 mmol/mol (8.0%) or lower than 48 mmol/mol (6.5%), with or without hypoglycemic symptoms. Exercise will be recommended for both groups and not a

part of the intervention. There will be no limitation in medication for hypertension, hyperuricemia and aspirin for prevention of cardiovascular disease. The will be social connected with the "line" social network within groups to encourage the compliance.

The dietitian will educate every participant for 30 minutes and will follow up the participants at the 2nd week and monthly for 3 months. The dietitian will follow up the patients every 3 months in person for 18 months and monthly telephone contact. The " line" social network within group provides sharing of appropriate food.

6. Outcome measurement

The primary outcomes are glycemic control (HbA1c, fasting and 2-hr glucose), dosage and types of hypoglycemic agents and insulin every 3 months for 18 months.

The secondary outcomes are:

(1)Biochemical markers: lipid profile (Cholesterol, triglyceride, HDL and LDL) and uric acid

(2)Endocrine markers: insulin(c-peptide for insulin uses) ,Apo-B and HOMA-IR, HOMA-beta cell function every 6 months for 18 months

(3) renal function: follow up renal function and microalbuminuria at 18 months

(4) blood pressure: every 3 months for 18 months

(5) body composition measurement: weight, BMI, waist circumference, hip circumference, and hip circumference and body fat composition every 3 months for 18 months

(6)functional assessment：3-m walk speed and grip strength every 3 months for 18months

(7)food recalls：2nd, 4th, 8th 12th weeks and every 3 months for 18 months

(8)compliance： the difficulty of compliance will be assessed at 2nd, 4th, 8th 12th weeks and every 3 months for 18 months

(9) other medication adjustment：the medication change, including lipid lowering agents, uricosuric agents。

(10) quality of life：WHOQOL-BREF and The 17-item version of the Chinese Diabetes Distress Scale (CDDS-17) were assesses at 3 months and 18 months

(11) atherosclerosis：carotid intimal thickness and ABI at 18 months

(12) diabetic retinopathy screening：follow up at 18 months

(13)cardiovascular disease： resting EKG at 18 months

(14) cancer screening：iFOBT for colon cancer screening and mammography for women

(15) fatty liver and gallstone: follow up abdominal echo at 18 month

(16) questionnaire for GERD：check Chinese GERDQ)[78] at the 3 and 18 months

(17) cognition and depression of the elderly：follow up Mini-State Mental Examination (MMSE) and Geriatric Depression Scale-4 (GDS-4) at 18 month

(18) physical activity: check Taiwan International Physical Activity Questionnaire at 3,6,12 and 18 months

(19) cognition and depression of the elderly：follow up Mini-State Mental Examination (MMSE) and Geriatric Depression Scale-4 (GDS-4) at 18 month

(20) physical activity: check Taiwan International Physical Activity Questionnaire at 3,6,12 and 18 months

Second year

Keep trait of the above intervention, outcome assessment and adverse event monitoring. Every participant will complete the 18-month follow up and data will be saved.

The items will be measured at the end of study (18 month) are:

(1) quality of life：WHOQOL-BREF

(2) atherosclerosis：carotid intimal thickness and ABI

(3) cataract and diabetic retinopathy：follow up at 18 months

8. Sample size estimation

According to a previous study, the estimated difference in the HbA1c reduction between the LCD and calorie-restricted diet (CRD) groups is 0.5%, with a standard deviation (SD) 0.408%. With a two-sided level of 5%, a power (1-ß) of 80%, and an assumed 20% loss to follow-up rate, the appropriate sample size was calculated to be 80 patients (20 patients in each group stratified by gender and BMI).

N = $\dfrac{\left(1+\dfrac{1}{m}\right)*(Z_\alpha+Z_\beta)^2\sigma^2}{d^2}$
考慮預期失訪率之樣本數<br>$\dfrac{N}{(1-p)^2}$

The estimation of sample size is 20 for a stratified cell. We will choose 30 if possible for a stratified cell because we use stratified random sample. According to the stratified layer by BMI and sex (BMI≦24 and >24; men and women). The total number will be 120, with control group and experimental group in parallel-designed.

9. Statistical analysis

The analysis will be performed using an intention-to-treat analysis; baseline or last

observations will be carried forward if the complete set of data for an individual is not available. The frequency or mean±SD will be presented for demographic data and single variant analysis. The repeated measures ANOVA wlll be conducted for different time group difference. The independent t tests or Wilcoxon rank tests will be used according to the normal distribution or not, for example, the different diet composition. A p-value <0.05 denoted a statistically significant difference.

Expected barriers and solutions

1. Recruiting study population in short term：recruitment from multiple systems, such as outpatient clinics, health check-up, dietitian clinic, poster announcement
2. Acceptance of low carbohydrate diet: prepared diet education pamphlet
3. Poor compliance：dietary monitoring and follow up biweekly to monthly, social media “line” for encouragement

Steps

First year

1. Recruitment of human resources: selection of research assistants with nutritional background
2. Research meeting: The research team will hold a meeting to discuss the research details and process, and determine the content, implementation location and pathways.
3. Determining the content of nutrition consultation: literature review, discussion with dietitians, setting food ingredient list, cooking recipes, self-monitoring recommendations, set up each nutrition consultation and tracking content
4. Print questionnaire, nutrition list, recipe, tracking manual
5. Assistant training: training assistant according to the above content and confirms that it can be executed correctly.
6. Recruitment of participants: recruitment from multiple systems, such as outpatient clinics, health check-up, dietitian clinic, poster announcement
7. Randomization to the experimental group or the control group .
8. Execution of intervention: execution according to the intervention plan for the experimental group and the control group respectively
9. Measurement of outcomes: data collection according to the established primary and secondary indicators
10. Adverse event monitoring: data collection according to the monitoring event
11. Data collection and input: input the above results into the computer

Second year

1. Execution of intervention: execution according to the intervention plan for the experimental group and the control group respectively
2. Measurement of outcomes: data collection according to the established primary and secondary indicators
3. Adverse event monitoring: data collection according to the monitoring event
4. Data collection and input: input the above results into the computer
5. Data Analysis: Analysis using package statistical software
6. Report writing

Execution flowchart

month	2	4	6	8	10	12	14	16	18	20	22	24
Recruitment of human resources												
Research meeting												
Identify nutrition consultation content												
Print questionnaire, nutrition list, recipe, tracking manual												
Assistant training												
Recruitment												
Randomization												
Intervention												
Outcome measurement												
Adverse event monitoring												
Data collection and input												
Data analysis												
Report writing												

(C) Expected work items, results and performance.

1. Expected work items to be completed

First year:

(1) Estimation of carbohydrate content in various diets
(2) Low carbohydrate diet content
(3) Low carbohydrate diet recipe
(4) Eat-out recommendation for low carbohydrate diet
(5) Self -monitoring methods for low carbohydrate diet
(6) Complete the recruitment, baseline assessment and randomization
(7) Use the “line” group to improve the content of diet control
(8) Diet compliance monthly
(9) Follow-up visit every 3 months
(10) Laboratory measurement and questionnaire assessment every 3 months
(11) Adverse events every 3 months

Second year：

(1) Diet compliance monthly for 18 months
(2) Follow-up visit every 3 months for 18 months
(3) Laboratory measurement and questionnaire assessment every 3 months for 18 months
(4) Adverse events monitoring every 3 months for 18 months
(5) Complete data entry and debugging
(6) Complete statistical analysis
(7) Completion of report writing

2. Expected contributions to academic research, national development and other applications

First year:

(1) The first study of low carbohydrate diet for our country
(2) Short-term effects of low carbohydrate diet on diabetes control
(3) Short-term effects of low carbohydrate diet on quality of life in diabetes
(4) Monitoring of adverse events of low carbohydrate diet

Second year:

(1) The long-term effect of low carbohydrate diet on diabetes control
(2) Long-term effects of low carbohydrate diet on quality of life in diabetes
(3) Based on this study result, increase sample size to analyze the effect on cardiovascular disease and mortality.

3. Expected training for the participating staff

First year

(1) Establishment of an educational model for low carbohydrate diet
(2) Creation of low carbohydrate diet recipe
(3) Eat-out recommendations for low carbohydrate diet
(4) Ways to improve compliance with low carbohydrate diets

Second year

(1) Low carbohydrate diet for the reduction of diabetes drugs
(2) The effect of low carbohydrate diet on diabetes control
(3) Satisfaction with low carbohydrate diet

4. Expected research results and performance (such as journal papers, conference papers, special books, technical reports, technology transfer, quality and quantity based performance)

First year

Low carbohydrate diet conference paper

Low carbohydrate diet journal paper

Second year

Low carbohydrate diet conference paper

Low carbohydrate diet journal paper

Low carbohydrate diet special book

References:

1. Ajala O, English P, Pinkney J.:Systematic review and meta-analysis of different dietary approaches to the management of type 2 diabetes. Am J Clin Nutr. 2013 Mar;97(3):505-16.
2. Standards of Medical Care in Diabetes 2007. Diabetic Care. 2007;30(supp1): S4-38.
3. Standards of Medical Care in Diabetes 2008. Diabetic Care. 2008;31(supp1): S12-54.
4. Standards of Medical Care in Diabetes 2011. Diabetic Care. 2011;34(supp1): S11-51.
5. Life with Diabetes: A Series of Teaching Outlines Paperback – November 19, 2009 By The Michigan Diabetes Research and Training Center.
6. Standards of Medical Care in Diabetes 2014. Diabetic Care. 2013;36(supp1): S12-56.
7. Standards of Medical Care in Diabetes 2014. Diabetic Care. 2014;37(supp1): S14-80.
8. Feinman RD, Pogozelski WK, Astrup A et al: Dietary carbohydrate restriction as the first approach in diabetes management: Critical review and evidence base. Nutrition. 2014 Jul 16.
9. Samaha FF, Iqbal N, Seshadri P, Chicano KL, Daily DA, McGrory J,Williams T, Williams M, Gracely EJ, Stern L. A low-carbohydrate as compared with a low-fat diet in severe obesity. N Engl J Med 2003;348:2074–81.
10. Stern L, Iqbal N, Seshadri P, Chicano KL, Daily DA, McGrory J,Williams M, Gracely EJ, Samaha FFThe effects of low-carbohydrate versus conventional weight loss diets in severely obese adults: one year follow-up of a randomized trial. Ann Intern Med 2004;140:778–85.
11. Westman EC, Yancy WS, Mavropoulos JC, Marquart M, McDuffie JR.:The effect of a low-carbohydrate, ketogenic diet versus a lowglycemic index diet on glycemic index diet on glycemic control in type 2 diabetes mellitus. Nutr Metab (Lond) 2008;5:36.
12. Wolever TM, Gibbs AL, Mehling C, Chiasson JL, Connelly PW, Josse RG, Leiter LA, Maheux P, Rabasa-Lhoret R, Rodger NW, et al. The Canadian Trial of Carbohydrates in Diabetes (CCD), a 1-y controlled trial of low-glycemic-index dietary carbohydrate in type 2 diabetes: no effect on glycated hemoglobin but reduction in C-reactive protein. Am J Clin Nutr 2008;87:114–25.
13. Haimoto H, Iwata M,Wakai K, Umegaki H. Long-term effects of a diet loosely restricting carbohydrates on HbA1c levels, BMI and tapering of sulfonylureas in type 2 diabetes: a 2-year follow-up study. Diabetes Res Clin Pract 2008 ;79:350–6.

14. Davis NJ, Tomuta N, Schechter C, Isasi CR, Segal-Isaacson CJ, Stein D, Zonszein J, Wylie-Rosett J. Comparative study of the effects of a 1-year dietary intervention of a low-carbohydrate diet versus a low- fatdiet on weight and glycemic control in type 2 diabetes. Diabetes Care 2009;32:1147–52.
15. Elhayany A, Lustman A, Abel R, Attal-Singer J, Vinker S. A low carbohydrate Mediterranean diet improves cardiovascular risk factors and diabetes control among overweight patients with type 2 diabetes mellitus: a 1-year prospective randomized intervention study. Diabetes Obes Metab 2010;12:204–9.
16. Yang WS Jr, Westman EC, McDuffie JR, Grambow SC, Jeffreys AS, Bolton J, Chalecki A, Oddone EZ. A randomized trial of a lowcarbohydrate diet vs orlistat plus a low-fat diet for weight loss. Arch Intern Med 2010;170:136–45.
17. Iqbal N, Vetter ML, Moore RH, Chittams JL, Dalton-Bakes CV, Dowd M, Williams-Smith C, Cardillo S, Wadden TA. Effects of a low-intensity intervention that prescribed a low-carbohydrate vs. a low-fat diet in obese. Obesity (Silver Spring) 2010;18:1733–8.
18. Yamada Y, Uchida J, Izumi H, Tsukamoto Y, Inoue G, Watanabe Y, Irie J, Yamada S:.A non-calorie-restricted low-carbohydrate diet is effective as an alternative therapy for patients with type 2 diabetes. Intern Med. 2014;53(1):13-9.
19. Tirosh A, Golan R, Harman-Boehm I, Henkin Y, Schwarzfuchs D, Rudich A, Kovsan J, Fiedler GM, Bl"uher M, Stumvoll M, Thiery J, Stampfer MJ, Shai I:Renal function following three distinct weight loss dietary strategies during 2 years of a randomized controlled trial. Diabetes Care. 2013 Aug;36(8):2225-32 20.
20. Santos FL, Esteves SS, da Costa Pereira A, Yancy WS Jr, Nunes JP. Systematic review and meta-analysis of clinical trials of the effects of low carbohydrate diets on cardiovascular risk factors. Obes Rev. 2012 Nov;13(11):1048-66.
21. Ruth MR, Port AM, Shah M, Bourland AC, Istfan NW, Nelson KP, Gokce N, Apovian CM: Consuming a hypocaloric high fat low carbohydrate diet for 12 weeks lowers C-reactive protein, and raises serum adiponectin and high density lipoprotein -cholesterol in obese subjects. Metablosim 2013 Dec;62(12):1779-87.
22. Larsen RN, Mann NJ, Maclean E, Shaw JE. The effect of high-protein, low-carbohydrate diets in the treatment of type 2 diabetes: a 12 month randomised controlled trial Diabetologia. 2011;54(4):731-40.
23. Guldbrand H1, Dizdar B, Bunjaku B, Lindström T, Bachrach-Lindström M, Fredrikson M, Ostgren CJ, Nystrom FH: In type 2 diabetes, randomisation to advice to follow a low-carbohydrate diet transiently improves glycaemic control compared with advice to follow a low-fat diet producing a similar weight loss. Diabetologia. 2012 Aug;55(8):2118-27.
24. Krebs JD1, Elley CR, Parry-Strong A, Lunt H, Drury PL, Bell DA, Robinson E,

Moyes SA, Mann JI: The Diabetes Excess Weight Loss (DEWL) Trial: a randomised controlled trial of high-protein versus high-carbohydrate diets over 2 years in type 2 diabetes. Diabetologia. 2012 Apr;55(4):905-14.
25. Dyson PA, Beatty S, Matthews DR An assessment of low-carbohydrate or low-fat diets for weight loss at 2 year's follow-up. Diabet Med. 2010 Mar;27(3):363-4.
26. Hussain TA, Mathew TC, Dashti AA, Asfar S, Al-Zaid N, Dashti HM. Effect of low-calorie versus low-carbohydrate ketogenic diet in type 2 diabetes. Nutrition. 2012 Oct;28(10):1016-21.
27. Centers for Disease Control and Prevention (CDC). Trends in intake of energy and macronutrients--United States, 1971-2000. MMWR Morb Mortal Wkly Rep. 2004 Feb 6;53(4):80-2.
28. J.S. Volek, M.L. Fernandez, R.D. Feinman, S.D. Phinney Dietary carbohydrate restriction induces a unique metabolic state positively affecting atherogenic dyslipidemia, fatty acid partitioning, and metabolic syndrome. Prog Lipid Res, 47 (2008), pp. 307–318.
29. A. Al-Khalifa, T.C. Mathew, N.S. Al-Zaid, E. Mathew, H.M. Dashti Therapeutic role of low-carbohydrate ketogenic diet in diabetes. Nutrition, 25 (2009), pp. 1177–1185.
30. M.C. Gannon, H. Hoover, F.Q. Nuttall Further decrease in glycated hemoglobin following ingestion of a LoBAG30 diet for 10 wk compared with 5 wk in people with untreated type 2 diabetes. Nutr Metab (Lond), 7 (2010), p. 64.
31. P.A. Dyson, S. Beatty, D.R. Matthews A low-carbohydrate diet is more effective in reducing body weight than healthy eating in both diabetic and non-diabetic subjects. Diabet Med, 24 (2007), pp. 1430–1435.
32. B.V. Howard, J.E. Manson, M.L. Stefanick, S.A. Beresford, G. Frank, B. Jones, et al. Low-fat dietary pattern and weight change over 7 y: the Women’s Health Initiative dietary modification trial. JAMA, 295 (2006), pp. 39–49.
33. R.C. Atkins. Dr. Atkins’ new diet revolution. Avon Books, New York (2002)
34. E.C. Westman, S.D. Phinney, J. Volek The new Atkins for a new you: the ultimate diet for shedding weight and feeling great forever. Simon & Schuster, New York (2010).
35. Gunnars, K. Low-Carb Diets – Healthy, but Hard to Stick to? In: Authority Nutrition. 2013. Available at http://AuthorityNutrition.com. Accessed September 6, 2014.
36. A. Belza, C. Ritz, M.Q. Sorensen, J.J. Holst, J.F. Rehfeld, A. Astrup Contribution of gastroenteropancreatic appetite hormones to protein-induced satiety. Am J Clin Nutr, 97 (2013), pp. 980–989.

37. R.D. Feinman, M.C. Vernon, E.C. Westman Low carbohydrate diets in family practice: what can we learn from an internet-based support group. Nutr J, 5 (2006), p. 26
38. J.W. Krieger, H.S. Sitren, M.J. Daniels, B. Langkamp-Henken Effects of variation in protein and carbohydrate intake on body mass and composition during energy restriction: a meta-regression 1. Am J Clin Nutr, 83 (2006), pp. 260–274.
39. R.D. Feinman, E.J. Fine "A calorie is a calorie" violates the second law of thermodynamics. Nutr J, 3 (2004), p. 9
40. R.D. Feinman, E.J. Fine Nonequilibrium thermodynamics and energy efficiency in weight loss diets. Theor Biol Med Model, 4 (2007), p. 27
41. Siri-Tarino, P.W., Sun, Q., Hu, F.B., and Krauss, R.M. Meta-analysis of prospective cohort studies evaluating the association of saturated fat with cardiovascular disease. Am J Clin Nutr. 2010; 91: 535–546.
42. Siri-Tarino, P.W., Sun, Q., Hu, F.B., and Krauss, R.M. Saturated fat, carbohydrate, and cardiovascular disease. Am J Clin Nutr. 2010; 91: 502–509
43. Weinberg, S.L. The diet-heart hypothesis: a critique. J Am Coll Cardiol. 2004; 43: 731–733.
44. Ravnskov, U., Rosch, P.J., Sutter, M.C., and Houston, M.C. Should we lower cholesterol as much as possible?. Bmj. 2006; 332: 1330–1332.
45. Yancy, W.S. Jr., Westman, E.C., French, P.A., and Califf, R.M. Diets and clinical coronary events: The truth is out there. Circulation. 2003; 107: 10–16
46. Teicholz, N. The big fat surprise. Why butter, meat & cheese belong in a healthy diet. Simon & Schuster, New York; 2014.
47. Kendrick, M. The great cholesterol con: the truth about what really causes heart disease and how to avoid it. John Blake, London; 2008.
48. Ravnskov, U. The cholesterol myths: exposing the fallacy that cholesterol and saturated fat cause heart disease. NewTrends Publishing, Inc., Washington, DC; 2000.
49. Taubes, G. Good calories, bad calories. Alfred A. Knopf, New York; 2007.
50. Jakobsen, M.U., O'Reilly, E.J., Heitmann, B.L., Pereira, M.A., Balter, K., Fraser, G.E. et al. Major types of dietary fat and risk of coronary heart disease: a pooled analysis of 11 cohort studies. Am J Clin Nutr. 2009; 89: 1425–1432.
51. Lin, J., Wu, P.H., Tarr, P.T., Lindenberg, K.S., St-Pierre, J., Zhang, C.Y. et al. Defects in adaptive energy metabolism with CNS-linked hyperactivity in PGC-1 alpha null mice. Cell. 2004; 119: 121–135.
52. Forsythe, C.E., Phinney, S.D., Feinman, R.D., Volk, B.M., Freidenreich, D., Quann, E. et al. Limited effect of dietary saturated fat on plasma saturated fat in the context of a low carbohydrate diet. Lipids. 2010; 45: 947–962.
53. Forsythe, C.E., Phinney, S.D., Fernandez, M.L., Quann, E.E., Wood, R.J., and

Bibus, D.M. Comparison of low fat and low carbohydrate diets on circulating fatty acid composition and markers of inflammation. Lipids. 2008; 43: 65–77.
54. Volek, J.S., Phinney, S.D., Forsythe, C.E., Quann, E.E., Wood, R.J., Puglisi, M.J. et al. Carbohydrate restriction has a more favorable impact on the metabolic syndrome than a low fat diet. Lipids. 2009; 44: 297–309.
55. Stratton, I.M., Adler, A.I., Neil, H.A., Matthews, D.R., Manley, S.E., Cull, C.A. et al. Association of glycaemia with macrovascular and microvascular complications of type 2 diabetes (UKPDS 35): prospective observational study. BMJ. 2000; 321: 405–412.
56. Turner, R.C. The U.K. Prospective Diabetes Study. A review. Diabetes Care. 1998; 21: C35–38.
57. Turner, R.C., Millns, H., Neil, H.A., Stratton, I.M., Manley, S.E., Matthews, D.R. et al. Risk factors for coronary artery disease in non-insulin dependent diabetes mellitus: United Kingdom Prospective Diabetes Study (UKPDS: 23). BMJ. 1998; 316: 823–828.
58. Jenkins, D.J., Kendall, C.W., McKeown-Eyssen, G., Josse, R.G., Silverberg, J., Booth, G.L. et al. Effect of a low-glycemic index or a high-cereal fiber diet on type 2 diabetes: a randomized trial. JAMA. 2008; 300: 2742–2753.
59. Feinman, R.D., Volek, J.S., and Westman, E. Dietary carbohydrate restriction in the treatment of diabetes and Mmtabolic syndrome. Clin Nutr Insight. 2008; 34: 1–5.
60. Barter, P.J., Ballantyne, C.M., Carmena, R., Castro Cabezas, M., Chapman, M.J., Couture, P. et al. Apo B versus cholesterol in estimating cardiovascular risk and in guiding therapy: Report of the thirty-person/ten-country panel. J Intern Med. 2006; 259: 247–258.
61. Dreon, D.M., Fernstrom, H.A., Campos, H., Blanche, P., Williams, P.T., and Krauss, R.M. Change in dietary saturated fat intake is correlated with change in mass of large low-density-lipoprotein particles in men. Am J Clin Nutr. 1998; 67: 828–836.
62. Dreon, D.M., Fernstrom, H.A., Williams, P.T., and Krauss, R.M. A very low-fat diet is not associated with improved lipoprotein profiles in men with a predominance of large, low-density lipoproteins. Am J Clin Nutr. 1999; 69: 411–418.
63. McLaughlin, T., Reaven, G., Abbasi, F., Lamendola, C., Saad, M., Waters, D. et al. Is there a simple way to identify insulin-resistant individuals at increased risk of cardiovascular disease?. Am J Cardiol. 2005; 96: 399–404.
64. Nielsen, J.V., Gando, C., Joensson, E., and Paulsson, C. Low carbohydrate diet in type 1 diabetes, long-term improvement and adherence: a clinical audit. Diabetol Metab Syndr. 2012; 4: 23.
65. Bernstein, R.K. Dr. Bernstein’s diabetes solution: the complete guide to achieving normal blood sugars. 4th ed. Little, Brown and Co, New York; 2011.

66. Saslow, L.R., Kim, S., Daubenmier, J.J., Moskowitz, J.T., Phinney, S.D., Goldman, V. et al. A randomized pilot trial of a moderate carbohydrate diet compared with a very low carbohydrate diet in overweight or obese individuals with type 2 diabetes mellitus or prediabetes. PLoS One. 2014; 9: e91027.
67. Yancy, W.S. Jr., Foy, M., Chalecki, A.M., Vernon, M.C., and Westman, E.C. A low-carbohydrate, ketogenic diet to treat type 2 diabetes. Nutr Metab (Lond). 2005; 2: 34.
68. Nielsen, J.V. and Joensson, E. Low-carbohydrate diet in type 2 diabetes. Stable improvement of bodyweight and glycemic control during 22 mo follow-up. Nutr Metab (Lond). 2006; 3: 22.
69. Nielsen, J.V., Jonsson, E., and Nilsson, A.K. Lasting improvement of hyperglycaemia and bodyweight: low-carbohydrate diet in type 2 diabetes—a brief report. Ups J Med Sci. 2005; 110: 69–73.
70. Boden, G., Sargrad, K., Homko, C., Mozzoli, M., and Stein, T.P. Effect of a low-carbohydrate diet on appetite, blood glucose levels, and insulin resistance in obese patients with type 2 diabetes. Ann Intern Med. 2005; 142: 403–411.
71. Gerstein, H.C., Miller, M.E., Byington, R.P., Goff, D.C. Jr., Bigger, J.T., Buse, J.B. et al. Effects of intensive glucose lowering in type 2 diabetes. N Engl J Med. 2008; 358: 2545–2559.
72. 江部 康二 糖尿病治療のための! 糖質制限食パーフェクトガイド 2013/8/9.
73. 江部 康二 主食を抜けば糖尿病は良くなる! 新版: 糖質制限食のすすめ 2014/3/14
