# Supplementary material for: Effect of a 90 g/day low-carbohydrate diet on glycaemic control, small, dense low-density lipoprotein and carotid intima-media thickness in type 2 diabetic patients: An 18-month randomised controlled trial
Source: PLoS One. 2020 Oct 5;15(10):e0240158. doi: 10.1371/journal.pone.0240158 (PMC7535044; doi:10.1371/journal.pone.0240158)
Supplement: Attachment — Submitted filename: PLos one response 20200327.docx [file pone.0240158.s004.docx]

---------- Forwarded message ---------

From: PLOS ONE <em@editorialmanager.com>

Date: 2019年2月25日(月) 午後7:33

Subject: PLOS ONE Decision: PONE-D-18-29713 - [EMID:16cda7a4b701eb18]

To: Wei Sheng Huang <starsambora@gmail.com>

PONE-D-18-29713

Effect of a 90 g/day low-carbohydrate diet on glycemic control, small dense low density lipoprotein, and carotid intima-media thickness in type 2 diabetes patients: an 18-month randomized controlled trial

PLOS ONE

Dear Dr. Huang,

Thank you for submitting your manuscript to PLOS ONE. After careful consideration, we have decided that your manuscript does not meet our criteria for publication and must therefore be rejected.

Specifically:

This decision was based not only on the reviewers' comments below, but also on the Editors' evaluation of the merits of your manuscript compared with those of the many others that we receive. This evaluation included consideration of the originality, design and results of the study, the composition of the manuscript, the overall significance of the work, and its interest to our readers.

Specifically, sample size calculation and analysis of the data appear to be inadequate as pointed by the reviewer with substantial statistical experience. This is a major flaw of the study. In addition, the language of the paper is difficult to understand and includes many errors. PLOS ONE does not copyedit accepted manuscripts, so the language in submitted articles must be clear, correct, and unambiguous.

The rejection does not preclude future reconsideration if you decide to pursue new submission. If you decide to do a new submission, we recommend that you seek independent editorial help. These services can be found on the web using search terms like “scientific editing service” or “manuscript editing service”.

Thank you for the opportunity to review your paper. We appreciate your interest in Plos One.

I am sorry that we cannot be more positive on this occasion, but hope that you appreciate the reasons for this decision.

Yours sincerely,

Elena Barengolts, MD

Academic Editor

PLOS ONE

Response: The sample size calculation was explained and the analysis of data was re-done. The English was edited again.

[Note: HTML markup is below. Please do not edit.]

Reviewers' comments:

Reviewer's Responses to Questions

Comments to the Author

1. Is the manuscript technically sound, and do the data support the conclusions?

The manuscript must describe a technically sound piece of scientific research with data that supports the conclusions. Experiments must have been conducted rigorously, with appropriate controls, replication, and sample sizes. The conclusions must be drawn appropriately based on the data presented.

Reviewer #1: Yes

Reviewer #2: Yes

Reviewer #3: No

--------------------------------------------------------------------------------

2. Has the statistical analysis been performed appropriately and rigorously?

Reviewer #1: Yes

Reviewer #2: I Don't Know

Reviewer #3: No

--------------------------------------------------------------------------------

3. Have the authors made all data underlying the findings in their manuscript fully available?

The PLOS Data policy requires authors to make all data underlying the findings described in their manuscript fully available without restriction, with rare exception (please refer to the Data Availability Statement in the manuscript PDF file). The data should be provided as part of the manuscript or its supporting information, or deposited to a public repository. For example, in addition to summary statistics, the data points behind means, medians and variance measures should be available. If there are restrictions on publicly sharing data—e.g. participant privacy or use of data from a third party—those must be specified.

Reviewer #1: Yes

Reviewer #2: Yes

Reviewer #3: Yes

--------------------------------------------------------------------------------

4. Is the manuscript presented in an intelligible fashion and written in standard English?

PLOS ONE does not copyedit accepted manuscripts, so the language in submitted articles must be clear, correct, and unambiguous. Any typographical or grammatical errors should be corrected at revision, so please note any specific errors here.

Reviewer #1: Yes

Reviewer #2: Yes

Reviewer #3: No

--------------------------------------------------------------------------------

5. Review Comments to the Author

Please use the space provided to explain your answers to the questions above. You may also include additional comments for the author, including concerns about dual publication, research ethics, or publication ethics. (Please upload your review as an attachment if it exceeds 20,000 characters)

Reviewer #1: I feel that this paper has been written very well, answers an important question in a methodically sound manner.

Information I feel is lacking includes:

1) The demographic information could be made a little clearer (perhaps personal opinion)

Response: The table 1was rewritten and demonstrated the demographic factors only.(line 210)

2) What support was offered to the patients in the intervention and control arms following the initial intervention throughout the 18 months - how can you explain the unusually high completion rates?

Response: The high completion rate was because these patients with chronic disease were routinely cared by their family physicians before enrolling into the study. They agreed with the arrangements of experimental blood test and follow-up visits with their routine appointments without any incentives.(line 314-319)

3) Could you share a breakdown of saturated fat and unsaturated fat intakes of both groups in addition to total fat intake - this could then be discussed in relation to markers such as sdLDL and other cardiovsacular markers

Response:

(a) The LCD group took significant more saturated fat (p < 0.05) and monounsaturated fat (p < 0.05) than the TDD group at most visits (line 216-218)
(b) (Table 2).(line 233)

(c)Conversely, the significantly greater intake of protein and fat, especially saturated fat, in the LCD group did not show adverse effects on LDL and sdLDL.(line 351-353)

4) I feel it could be useful to link the fact that there was no statistically significant difference in weight loss between groups to the fact that there was no significant difference in calorie intake between groups. This is often a debated topic in terms of what creates the weight loss - is it low carb or is it just total calories.

Response: By different statistic methods, the weight loss was significantly lower in the LCD group than the TDD group.

(a) Comparing with the baseline, weight, BMI, and the waist, hip, and thigh circumferences at 18 months were significantly decreased in the LCD group, whereas only the weight, BMI, and thigh circumference were significantly decreased in the TDD group (p < 0.05). The decrease of weight, waist circumference and hip circumference from the baseline to 18-months was more significant in the LCD group comparing with that of the TDD group (p < 0.05) (line 299-304)
(b) (Table 3).(line 254-255)
(c) Although the net weight reduction was about 3 kg in the LCD group, the 18-month changes from baseline were significantly reduced in weight and waist and hip circumference in the LCD group compared to the TDD group. These findings suggest that the remodeling of fat composition existed in a small weight reduction among patients with poorly controlled type 2 DM. Besides, the small reduction in weight resulted in a significant blood pressure reduction and normalization of ALT (line 338-343).

From my perspective those were the main points, but I felt it was a well written paper with a well considered and explained methodology

Reviewer #2: Minor revisions

1. The uniqueness of the diet is in the fact that carb restriction was moderate as opposed to severe (<50g) or small (~150g/d). Emphasize this in your discussion and Abstract. Perhaps include in the paper title “…moderate (90g/d)…”

Response: In order to determine the long-term effect on glycemic control, lipids and atherosclerosis of moderate LCD on type 2 DM, we conducted an 18-month randomized clinical trial.(line 93-94)

2. The major problem appears to be the concept of standard “Calorie restricted diet”. CRD usually is aimed at body weight reduction and expected to decrease 500 cal/day to achieve that. In the study more than 20% of participants were not obese and some needed to increase their calories. Please do analysis of the Subgroup of “Obese”. A simple comparison of data (t-test) at the end of the study probably is the most important.

Response:

(a) Because of too many parameters in this study, we didn’t do subgroup analysis.
(b) We have re-done the statistics. The independent t-test was used to compare the differences or changed difference between two groups. The paired t-test was conducted to compare the 18-month data from that at the baseline within groups. The analysis of generalized estimating equation (GEE) parameters estimated the empirical standard error estimates for the time-group difference over the 18 months. All analyses were conducted using SAS 9.4 (2013) statistic software package. A p-value < 0.05 was deemed statistically significant. (line 197-203)
3. In the Methods: explain how often each group had meeting and with whom. Explain all additional support (phone, online, etc) that was provided for each group.

Response:

The patients were assessed by the research physician, the research nurse, and the dietitian at the first visit to record current medication use and compliance. Additionally, patients were instructed to keep a food diary for 3 days. Patients who complied with this requirement were evaluated by the first assessment and randomized by the research assistant after the first assessment. Individual dietary instruction was given by the research dietitian for each randomized group. Motivation group classes were arranged for both groups separately. (line 162-167)

All patients met with the research nurse every 3 months and were arranged with the clinic visit and reminded by phone calls (line 183-184)

4. Was there an incentive (payment, food, present, ect) to achieve high study completion rate?

Response: The high completion rate was because these patients with chronic disease were routinely cared by their family physicians before enrolling into the study. They agreed with the arrangements of experimental blood test and follow-up visits with their routine appointments without any incentives.(line 316-319)

5. Abstract: Add how many carbs in CRD group? How many calories each group consumed? In the Results section provide actual changes in each group for HbA1c, fasting glucose, etc and state p<0.05 for all. You can place all parameters with significant changes together and state p<0.05 for all. The state that there were no other significant differences for other parameters.

Responese:

(a) A total of 85 (92.4%) patients completed 18 months of the trial. At the end of the study, the LCD and the TDD group took daily carbohydrate of 88.0±29.9 g and 151.1±29.8 g separately (p < 0.05).And the daily calorie intake was not significantly different in the LCD and the TDD group at the 18 months (5984.0±1171.1 KJ vs 6145.5±1048.5 KJ) (p > 0.05).(line 33-36)
(b) The 18-month change from baseline were statistically significant for the HbA1c (-1.6 vs -1.0%), 2-hr glucose (-5.24 vs -1.04 mmol/L), weight (-2.8 vs -0.7 kg), waist circumference (-5.7 vs -1.9 cm), hip circumference (-6.0 vs -2.9 cm), alanine aminotransferase (ALT) (-0.13 vs -0.03µkat/L) and blood pressure(-8.3/-5.0 vs 1.6/2.5 mmHg) between the LCD and TDD groups (p<0.05). The 18-month change from baseline was not significantly different in MES, lipid profiles, sdLDL, serum creatinine, microalbuminuria , and carotid IMT between groups. (line 37-42)
6. Line (L)-60: Do not understand, explain what you mean: “The insulin resistance preceding metabolic syndrome and the common pathways for type 2 diabetes mellitus (DM) are lifestyle diseases that initiate a cascade of cardiovascular diseases and frailty.“

Response: This part was omitted.

7. L-75: Do not understand, explain what you mean: “However, the quality (size and density) rather than the quantity of LDL particles with carbohydrate metabolism has been characterized.”

Response: LDL is structurally heterogenous and is further categorized based on size. DM is associated with small dense LDL (sdLDL) particles that are associated with an increase in cardiovascular risk. (line 83-85)

8. L-65: “low-carbohydrate diets (LCDs)” Provide definition LCD, for example: LCD usually defined as …-…g/day”.

Response: low carbohydrate diet (LCD) (carbohydrate < 130 g/day or < 26% of total energy intake) (line 64-65)

9. L66: “short-term improvements in glycemic control” Provide range for “short-term”, for example ‘3-12 months’

Response: short-term (3-6 month) (line 66)

10. L-68; “longer periods” provide range, for example 12-24 months or 1-3 years.

Response: longer periods (1-2 years) (line 68)

11. Table-1: Explain meaning: “Types of diabetic medications”, “Self-reported carbohydrate”-g/day?

Response: types (categories) of diabetic medications (line 142-143)

Self-reported carbohydrate was omitted due to too many items.

12. Table 1: needs caption to explain all abbreviations including CRD, LCD, OHA, MES, etc. Also, under the Table provide conversion formula for kJ into calories.

Response:

(a) new table 1 with explanations of abbreviations (line 210)
(b) 1 KJ=0.239 Calorie (line 238)
13. Table 2: needs to be self-explanatory. Explain “Slope”

Response: This part was omitted and changed to Figure 2.

14. Table 2: p-value provided is difficult to understand for a clinician. Include simple statistics (t-test or similar) for between groups differences at the end (18 mo), this can be added as * for 18mo data.

Response: The table 2 was re-done to table 3 (line 254).

Reviewer #3: As stated by the authors, the goal of this study is to evaluate the effect of a 90 g/d low carbohydrate diet (LCD) on glycemic control, small dense low-density lipoprotein (sdLDL), and carotid intima-media thickness (IMT) in type 2 diabetes patients over 18 months. The study design is a two-arm randomized trial (LCD versus the other arm being the Calorie reduced diet-CRD) over 18 months of follow-up. The study primary endpoints are the glycemic control status (HbA1c, 124 fasting and 2-h glucose) and the change in the medication effect score (MES). The secondary outcomes are the lipid profile, sdLDL, serum creatinine, microalbuminuria, and carotid IMT.

The statistical analysis was performed using the intention-to-treat approach coupled with GEE based models to adjust for the within-patient association in the data, and last observations carried forward (LOCF) for attrition.

The study design is reasonably well described even though there are some confusions on the assumptions used for the sample size/power calculation. For example, on line 118, it is stated “According to a previous study [21], the estimated difference in the HbA1c reduction between the LCD and calorie-restricted diet (CRD) groups is 0.5%, with a standard deviation (SD) 0.408%.”, but it is unclear whether the stated difference (0.5%) is relative or absolute. This should be clarified.

More importantly, even though a GEE-based model is used for the longitudinal analysis, the linear straight line model imposed on the mean structure is very restrictive. Assume a straight line may mask non constant changes occurring between the baseline and 18 months of follow-up. For example, it could be the case that the mean for one of the endpoints (eg, HbA1c) only changes between the baseline and 3 months and remains constant thereafter. For such important characteristics of the average profile may not be assessed if the assumed model is too smooth. Don’t get me wrong here, the straight line model may well be appropriate to describe how the mean responses (eg, HbA1c) evolve over time, but this should be formally evaluated. An easy approach to evaluate the straight line model under the GEE framework (no data likelihood is assumed) is to consider the unstructured mean model that treats Time (Baseline, 3m, 6m, 12m, 15m and 18m) as a categorical variable, and use mean contrasts imposing a constant change across time. And then perform a Wald test to evaluate whether the imposed contrasts involving mean parameters are consistent with data. Obviously if you decide to use a likelihood-based model such as mixed model, a likelihood ratio test can also be used.

Another important issue is that of attrition which is addressed using the last observation carried forward analysis (line 202). This approach has well known limitations in that it artificially reduces the within-subject variability in the data, due to the constant profile being imposed from time of dropout to the end of study. Albeit the fact that only a small portion of subjects dropped out from this study, the authors may consider using the available case analysis approach. It is worth mentioning that the available case analysis under GEE may still be biased if missingness is not completely at random (Indeed, the original GEE-based model analysis is only valid under the most stringent condition of dropout mechanism being completely at random). If there are suspicions that the dropout mechanism is at random, the authors may consider the weighted GEE analysis to accommodate attrition. Another analytical approach that produces valid inferences under the MAR mechanism is the multiple imputation approach followed by analysis of individual data sets and summary of the estimates across the imputed data sets.

My major concern is that of the write-up of this manuscript. There are too many sentences that are not optimally constructed and are often questionable. I am given below, in addition to specific comments, few instances of these problems which by no means should be considered exhaustive.

Response:

(a) The difference is absolute.(line121)
(b) Statistics was re-done as the following:

The analysis was performed using an intention-to-treat analysis. The independent t-test was used to compare the differences or changed difference between two groups. The paired t-test was conducted to compare the 18-month data from that at the baseline within groups. The analysis of generalized estimating equation (GEE) parameters estimated the empirical standard error estimates for the time-group difference over the 18 months. All analyses were conducted using SAS statistic software package. A p-value < 0.05 was deemed statistically significant.(line 197-203)

Some specific suggestions/questions:

Line 32 (abstract results); The statement is confusing? “A total of 85 (92.4%) patients completed 18 months of the trial. The HbA1c, fasting glucose, and 2-hr glucose reduction were significantly greater for the LCD group from baseline to 18 months (p<0.05, p<0.05, p<0.001, respectively). The MES and number of diabetic medications were significantly lower for LCD groups from baseline to 18 months (all p<0.05).” what is being evaluated here? Is it the change from baseline? Or these responses (HbA1c, fasting glucose, and 2-hr glucose reduction) are also being evaluated at baseline. From the way it is written it appears as if the two groups were compared at each of the study time points. Because this is a longitudinal study, I would expect the changes from the baseline to be of interest.

Response: The abstract result was re-written:

Results

A total of 85 (92.4%) patients completed 18 months of the trial. At the end of the study, the LCD and the TDD group took daily carbohydrate of 88.0±29.9 g and 151.1±29.8 g separately (p < 0.001). And the daily calorie intake was not significantly different in the LCD and the TDD group at the 18 months (5984.0±1171.1 KJ vs 6145.5±1048.5 KJ) (p > 0.05). The 18-month change from baseline were statistically significant for the HbA1c (-1.6 vs -1.0%), 2-hr glucose (-5.24 vs -1.04 mmol/L), weight (-2.8 vs -0.7 kg), waist circumference (-5.7 vs -1.9 cm), hip circumference (-6.0 vs -2.9 cm), alanine aminotransferase (ALT) (-0.13 vs -0.03µkat/L) and blood pressure(-8.3/-5.0 vs 1.6/2.5 mmHg) between the LCD and TDD groups (p<0.05). The 18-month change from baseline was not significantly different in MES, lipid profiles, sdLDL, serum creatinine, microalbuminuria , and carotid IMT between groups.(line 32-42)

line 89: change “…or who were obese with type 2 DM.” to “…or an abnormally elevated BMI with type 2 DM.”

Response: ..with either a normal BMI or an abnormally elevated BMI.(line 96)

Line 102: change “…were diagnosed with diabetes ≥1 year ago…” to “…were diagnosed with diabetes for more than a year …”

Response: were diagnosed with diabetes for more than one year (line 107)

Lines 104-106: change “The patients identified for inclusion were referred by physicians from outpatient clinics at a medical center and were verified by a research assistant conducting the screening.” to “Potential study participants were referred by physicians from outpatient clinics at a medical center and screened by a research assistant.”

Response: Potential study participants were referred by physicians from outpatient clinics at the medical center and screened by a research assistant. (line109-110)

Line 136-138: Change “…the MES was calculated as 1.5×2 (mg)/8 (mg)+1.5×1500 (mg)/3000 (mg)=1.125. A higher MES value denoted a greater use of medication.” To “…the MES was calculated as 1.5×2 (mg)/8 (mg)+1.5×1500 (mg)/3000 (mg)=1.125, with higher values indicating a greater use of medication.”

Response: the MES was calculated as 1.5×2 (mg) / 8 (mg) + 1.5 × 1500 (mg) / 3000 (mg) = 1.125, with higher values indicating a greater use of medication.(line 139-140)

Lines 163-165: Change “The patients were assessed by the research physician, the research nurse, and the dietitian for the first visit to determine current medication and compliance; additionally, they were instructed to keep a food diary for 3 days.” to “The patients were assessed by the research physician, the research nurse, and the dietitian at the first visit to record current medication use and compliance. Additionally, patients were instructed to keep a food diary for 3 days.”

Response: The patients were assessed by the research physician, the research nurse, and the dietitian at the first visit to record current medication use and compliance. Additionally, patients were instructed to keep a food diary for 3 days. (line162-164)

Lines 165-168: Change “If the patients complied with the requirement to keep a food diary, randomization was performed by the research assistant after the first assessment, using Taves covariate-adaptive randomization, stratified using gender and BMI (<24 and ≥24).” to “Patients who complied with this requirement were randomized after the first assessment, using the Taves covariate-adaptive randomization scheme, stratified by gender and BMI (<24 and ≥24).

Response:

(a) Study design:

This was a single center, parallel-designed, randomized controlled trial, allocated with Taves covariate-adaptive randomization and stratified by sex and BMI (<24 and ≥24) (line 119-120)

(b) Patients who complied with this requirement were evaluated by the first assessment and randomized by the research assistant after the first assessment. (line 164-166)

Line 181: change “… for obese subjects with a BMI >24…” to “…for overweight/obese subjects (BMI >24)…”

Response: for overweight/obese subjects (BMI > 24) (line 176-177)

Lines 205-206: Give the working correlation of the GEE model.

Response: The analysis of generalized estimating equation (GEE) parameters estimated the empirical standard error estimates for the time-group difference over the 18 months. (line200-201)

Line 207: Cite SAS.

Response: SAS 9.4 (2013) (line 202)

Lines 207-208: change “A p-value <0.05 denoted a statistically significant difference.” To “A p-value <0.05 was deemed statistically significant.”

Response: A p-value < 0.05 was deemed statistically significant.(line202-203)

Lines 298-305. Rewrites these sections; what do you mean by “…did not significantly differ between the groups…” what does differ mean here? Temporal Change or difference at specific time point?

As I mentioned earlier, there are just too many sentences that have issues and clearly need to be rewritten.

Response: Changes in average carotid intimal thickness was re-written.

Comparing with the baseline, the average carotid IMT at 18 months increased for the TDD group (p < 0.05) and was stationary for the LCD group (p > 0.05). However, the 18-month change from baseline in the average carotid IMT was not significantly different between the two groups (p > 0.05) (Table 3) (line 285-289).

Changes in insulin and insulin resistance was omitted due to too many items.

--------------------------------------------------------------------------------

6. If you would like your identity to be revealed to the authors, please include your name here (optional).

Your name and review will not be published with the manuscript.

Reviewer #1: Rosemary Huntriss

Reviewer #2: Academic editor

Reviewer #3: (No Response)

[NOTE: If reviewer comments were submitted as an attachment file, they will be attached to this email and accessible via the submission site. Please log into your account, locate the manuscript record, and check for the action link "View Attachments". If this link does not appear, there are no attachment files to be viewed.]
