# Supplementary material for: Effect of a 90 g/day low-carbohydrate diet on glycaemic control, small, dense low-density lipoprotein and carotid intima-media thickness in type 2 diabetic patients: An 18-month randomised controlled trial
Source: PLoS One. 2020 Oct 5;15(10):e0240158. doi: 10.1371/journal.pone.0240158 (PMC7535044; doi:10.1371/journal.pone.0240158)
Supplement: Attachment — Submitted filename: Response to Reviewers.docx [file pone.0240158.s006.docx]

PONE-D-20-08376R1

Effect of a 90 g/day low-carbohydrate diet on glycaemic control, small, dense low-density lipoprotein and carotid intima-media thickness in type 2 diabetic patients: an 18-month randomised controlled trial

PLOS ONE

Please respond to the minor comments from Reviewer #1.

Please submit your revised manuscript by Sep 13 2020 11:59PM. If you will need more time than this to complete your revisions, please reply to this message or contact the journal office at plosone@plos.org. When you're ready to submit your revision, log on to https://www.editorialmanager.com/pone/ and select the 'Submissions Needing Revision' folder to locate your manuscript file.

Please include the following items when submitting your revised manuscript:

•A rebuttal letter that responds to each point raised by the academic editor and reviewer(s). You should upload this letter as a separate file labeled 'Response to Reviewers'.

•A marked-up copy of your manuscript that highlights changes made to the original version. You should upload this as a separate file labeled 'Revised Manuscript with Track Changes'.

•An unmarked version of your revised paper without tracked changes. You should upload this as a separate file labeled 'Manuscript'.

[Note: HTML markup is below. Please do not edit.]

Reviewers' comments:

Reviewer's Responses to Questions

Comments to the Author

1. If the authors have adequately addressed your comments raised in a previous round of review and you feel that this manuscript is now acceptable for publication, you may indicate that here to bypass the “Comments to the Author” section, enter your conflict of interest statement in the “Confidential to Editor” section, and submit your "Accept" recommendation.

Reviewer #1:?(No Response)

2. Is the manuscript technically sound, and do the data support the conclusions?

The manuscript must describe a technically sound piece of scientific research with data that supports the conclusions. Experiments must have been conducted rigorously, with appropriate controls, replication, and sample sizes. The conclusions must be drawn appropriately based on the data presented.

Reviewer #1:?Yes

3. Has the statistical analysis been performed appropriately and rigorously?

Reviewer #1:?(No Response)

4. Have the authors made all data underlying the findings in their manuscript fully available?

The PLOS Data policy requires authors to make all data underlying the findings described in their manuscript fully available without restriction, with rare exception (please refer to the Data Availability Statement in the manuscript PDF file). The data should be provided as part of the manuscript or its supporting information, or deposited to a public repository. For example, in addition to summary statistics, the data points behind means, medians and variance measures should be available. If there are restrictions on publicly sharing data—e.g. participant privacy or use of data from a third party—those must be specified.

Reviewer #1:?Yes

5. Is the manuscript presented in an intelligible fashion and written in standard English?

PLOS ONE does not copyedit accepted manuscripts, so the language in submitted articles must be clear, correct, and unambiguous. Any typographical or grammatical errors should be corrected at revision, so please note any specific errors here.

Reviewer #1:?Yes

6. Review Comments to the Author

Please use the space provided to explain your answers to the questions above. You may also include additional comments for the author, including concerns about dual publication, research ethics, or publication ethics. (Please upload your review as an attachment if it exceeds 20,000 characters)

Reviewer #1:?Minor comments

Table 1, it is best to provide symbol n (%) in the table. It may difficult for readers to identify which n(%) and mean (sd). Likewise the statistical tests to be denoted with symbol i.e. *, + etc

Answer: Thank you very much for your comments. N(%) and mean(SD) were provided in the table 1 . The statistical tests were denoted with symbol *.

(2)	*p < 0.05: significant difference between the groups by independent t-test for data of mean ± SD and by chi-square test (and Fisher’s exact test if n< 5) for data of number (%) (line 233).

Table 2, for the multiple comparison, were adjustment made to the p value? If not, the reason to be clearly stated in the statistical analysis section.

Answer: Thank you very much for your comments.

A p-value < 0.05 was deemed statistically significant for the primary end points (HbA1c, fasting glucose, 2-h glucose and the change in the MES) in the present trial. It is noted that the analyses were applied for the secondary end points using the p-value without adjustment for multiple comparison in the present trial.(line 218-221)

7. PLOS authors have the option to publish the peer review history of their article (what does this mean?). If published, this will include your full peer review and any attached files.

If you choose “no”, your identity will remain anonymous but your review may still be made public.

Do you want your identity to be public for this peer review? For information about this choice, including consent withdrawal, please see our Privacy Policy.

Reviewer #1:?No

?

[NOTE: If reviewer comments were submitted as an attachment file, they will be attached to this email and accessible via the submission site. Please log into your account, locate the manuscript record, and check for the action link "View Attachments". If this link does not appear, there are no attachment files.]

While revising your submission, please upload your figure files to the Preflight Analysis and Conversion Engine (PACE) digital diagnostic tool,?https://pacev2.apexcovantage.com/. PACE helps ensure that figures meet PLOS requirements. To use PACE, you must first register as a user. Registration is free. Then, login and navigate to the UPLOAD tab, where you will find detailed instructions on how to use the tool. If you encounter any issues or have any questions when using PACE, please email PLOS at?figures@plos.org. Please note that Supporting Information files do not need this step.
